# Structural and Mechanistic Insight into DNA Unwinding by *Deinococcus radiodurans* UvrD

**DOI:** 10.1371/journal.pone.0077364

**Published:** 2013-10-15

**Authors:** Meike Stelter, Samira Acajjaoui, Sean McSweeney, Joanna Timmins

**Affiliations:** 1 Structural Biology Group, European Synchrotron Radiation Facility, Grenoble, France; 2 University Grenoble Alpes, Institut de Biologie structurale, Grenoble, France; 3 Centre National de la Recherche Scientifique, Institut de Biologie structurale, Grenoble, France; 4 Commissariat à l’énergie atomique et aux énergies alternatives, Département du Science du Vivant, Institut de Biologie structurale, Grenoble, France; Saint Louis University, United States of America

## Abstract

DNA helicases are responsible for unwinding the duplex DNA, a key step in many biological processes. UvrD is a DNA helicase involved in several DNA repair pathways. We report here crystal structures of *Deinococcus radiodurans* UvrD (*dr*UvrD) in complex with DNA in different nucleotide-free and bound states. These structures provide us with three distinct snapshots of *dr*UvrD in action and for the first time trap a DNA helicase undergoing a large-scale spiral movement around duplexed DNA. Our structural data also improve our understanding of the molecular mechanisms that regulate DNA unwinding by Superfamily 1A (SF1A) helicases. Our biochemical data reveal that *dr*UvrD is a DNA-stimulated ATPase, can translocate along ssDNA in the 3′-5′ direction and shows ATP-dependent 3′-5′, and surprisingly also, 5′-3′ helicase activity. Interestingly, we find that these translocase and helicase activities of *dr*UvrD are modulated by the ssDNA binding protein. Analysis of *dr*UvrD mutants indicate that the conserved β-hairpin structure of *dr*UvrD that functions as a separation pin is critical for both *dr*UvrD’s 3′-5′ and 5′-3′ helicase activities, whereas the GIG motif of *dr*UvrD involved in binding to the DNA duplex is essential for the 5′-3′ helicase activity only. These special features of *dr*UvrD may reflect its involvement in a wide range of DNA repair processes *in vivo*.

## Introduction

Many biological processes, such as DNA replication, transcription, recombination or repair, require access to the genetic information hidden within the duplex DNA of the genome and for this purpose the double-stranded DNA (dsDNA) needs to be transiently unwound. A diverse set of enzymes, known as DNA helicases, is responsible for catalyzing this process [1,2]. DNA helicases are ubiquitous enzymes and many different helicases are found in a single cell due to the diversity of structures adopted by duplexed DNA. Helicases are a subset of the translocase enzyme family that share a number of conserved signature motifs responsible for either NTP binding and hydrolysis, DNA binding or for coupling these two processes. Based on primary structure analyses and extensive biochemical studies, six superfamilies of helicases have so far been described, each of which possesses a different set of conserved signature motifs [3,4]. Three of these superfamilies (SF1, SF2 and SF6) have been further classified according to their polarity 3′-5′ (type A) or 5′-3′ (type B) [4].

UvrD is classified as a SF1A helicase [3] and plays important functions in DNA replication [5], recombinational repair [6-8], methyl-directed mismatch repair [9] and nucleotide excision repair [10]. UvrD consists of two RecA-like domains (1A and 2A) that are responsible for nucleotide binding and hydrolysis and two additional domains (1B and 2B) that are involved in dsDNA binding. UvrD has been shown to translocate along single-stranded DNA (ssDNA) as a monomer, while a number of studies indicate that oligomerization, and notably dimerization, of UvrD is required for helicase activity [11-15]. Over the past 15 years, several crystal structures of SF1A helicases have been determined. In 1996, the structure of *Geobacillus stearothermophilus* PcrA (*gs*PcrA) was solved in its apo form [16] and in 1997, the first crystal structure of *Escherichia coli* Rep helicase complexed with ssDNA was solved [17] providing the first insights into the interaction of the protein with DNA. Subsequently, the structures of *gs*PcrA in complex with 3′-tailed DNA consisting of a 10 base pair DNA duplex and a seven base single-stranded 3′-tail were determined in apo- and AMPPNP-bound forms [18] and in 2006, several structures of *E. coli* UvrD (*ec*UvrD) bound to 3′-tailed DNA were determined revealing the details underlying DNA unwinding by SF1A helicases [19]. These structures led to the proposal of a combined wrench-and-inchworm mechanism for DNA unwinding [19,20]. In this model, a rotational movement regulated by ATP binding and hydrolysis acting as the ‘engine’ is combined with alternate tight and loose interactions at four protein-DNA contact points to produce a highly coordinated unidirectional movement along DNA. 

In the radiation-resistant bacterium, *Deinococcus radiodurans*, unlike in *E. coli*, UvrD is involved in diverse DNA repair pathways [7]. In particular, UvrD has been shown to play a central role in double-strand break (DSB) repair and reconstitution of the genome following chromosome fractionation [7]. In *E. coli*, the RecQ, RecD and Helicase IV enzymes participate in DSB repair while in *D. radiodurans*, these three helicases have been shown to be dispensable [7]. The involvement and importance of a helicase in a given cellular pathway are not conserved from one bacterium to another.

Here we present crystal structures of full-length and a C-terminally truncated construct of *D. radiodurans* UvrD (*dr*UvrD) in complex with 3′-tailed dsDNA. Our structures obtained in apo- and AMPPNP bound states provide us with several snapshots of this essential cellular process and reveal a large-scale spiral movement of UvrD around the duplexed DNA. Our structural data and biochemical analysis of wild-type and mutant *dr*UvrD support the previously proposed wrench-and-inchworm model and provide further insight into the local conformational changes associated with DNA unwinding. A structural comparison of *dr*UvrD with its *E. coli* homologue reveals that most of the differences reside in the inter-domain contacts and the ssDNA binding pocket and gating mechanism. Our biochemical studies reveal that *dr*UvrD is an active DNA-stimulated ATPase that also possesses ATP-dependent translocase and helicase activities. Further investigations of these *in vitro* activities demonstrated that *dr*UvrD translocates along ssDNA with a biased 3′-5′ directionality but, despite belonging to the SF1A protein family, can unwind duplexed DNA in both the 3′-5′ and 5′-3′ directions. Interestingly, we find that these translocase and bipolar helicase activities of *dr*UvrD are modulated by the ssDNA binding protein (SSB). 

## Materials and Methods

### Cloning, expression and purification of drUvrD and drSSB

**Figure 1:**
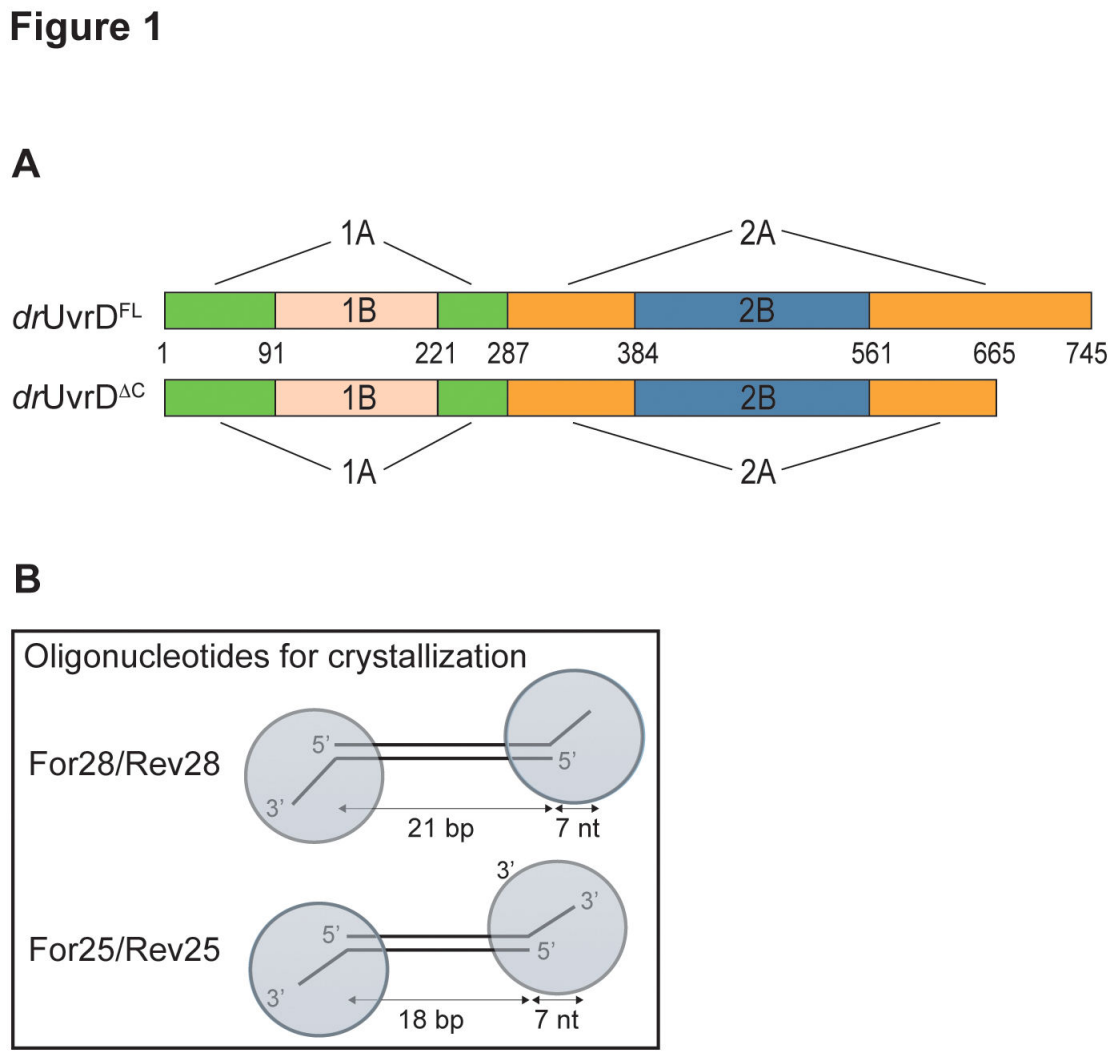
Domain organization of *dr*UvrD and structure of the various DNA oligonucleotides used for crystallization. A. Schematic representation of the domain structures of *dr*UvrD^FL^ and *dr*UvrD^∆C^. B. Structure of DNA oligonucleotides used for crystallization with *dr*UvrD^FL^ and *dr*UvrD^∆C^. The circles represent UvrD bound to the DNA as observed in our crystal structures.

Full-length *dr*UvrD (*dr*UvrD^FL^) and a truncated construct of *dr*UvrD (*dr*UvrD^∆C^), missing residues 666-745 (Figure 1A), were cloned into pET151d (Invitrogen). *dr*UvrD^FL^ mutants were prepared with the QuikChange mutagenesis kit (Agilent Technologies). All constructs were expressed in BL21 (DE3) cells. Protein expression was induced by 1 mM IPTG at 15°C overnight. Cells were lysed by sonication and the protein was purified by Ni affinity chromatography (Macherey-Nagel) in 50 mM Tris-HCl pH 8.0, 150 mM NaCl, 5% glycerol and 5 mM MgCl_2_, followed by His-tag cleavage with TEV protease and dialysis to remove the imidazole used for eluting the protein from the Ni column. The protein was further purified on a HiTrap Heparin column (GE Healthcare) and eluted in 50 mM Tris-HCl pH 8.0, 400 mM NaCl, 0.1 mM EDTA, 1 mM DTT, 5 mM MgCl_2_ and 5% glycerol. The protein was concentrated to 7-8 mg/ml and stored at -80°C. *Deinococcus radiodurans* SSB (*dr*SSB) was cloned into pET151d (Invitrogen) for expression with a cleavable N-terminal His-tag and expressed in BL21 (DE3) Star at 20°C overnight. *dr*SSB was purified on Ni-NTA (Qiagen) followed by a size-exclusion chromatography (Superdex 75 10/300 GL) in 50 mM Tris-HCl pH 8.0 and 100 mM NaCl and was stored at -80°C.

### DNA oligonucleotides

All DNA oligonucleotides used in this study were purchased from Eurofins-MWG and their sequences are presented in Table S1. The DNA used for co-crystallization with *dr*UvrD^∆C^ was composed of For25 and its complementary strand Rev25, while *dr*UvrD^FL^ was co-crystallized with DNA formed by For28 and Rev28 (Figure 1B). Annealed DNAs were purified by anion exchange chromatography (MonoQ; GE Healthcare). HPLC-purified oligonucleotides, For25-21F and Rev25-21F, containing a fluorescein-derivatized thymine (Fluo-dT) at position 21 were simply annealed prior to crystallization trials. For the helicase and DNA binding assays, the DNA substrates were made of a 25mer oligonucleotide containing a Fluo-dT at position 12 (H1T12) and a complementary oligonucleotide containing no extension (H4), a 15 nucleotide (nt) or a 7nt polydT ssDNA extension at either the 3′ (H3-15 or H3-7) or 5′ end (H5-15 or H5-7). For the streptavidin-displacement assay, the 3′-tailed DNA substrate consisted of a 3′-fluorescein labeled 25mer oligonucleotide (H1-3F) annealed to its complementary oligonucleotide with a 25nt polydT ssDNA extension at its 3′ end and containing a biotin conjugated thymine in position 49 (H3-25-B49), while the 5′-tailed DNA substrate was composed of a 5′-FAM labeled 25mer oligonucleotide (H1-5FAM) annealed to its complementary oligonucleotide with a 25nt polydT ssDNA extension at its 5′ end and containing a biotin conjugated thymine in position 2 (H5-25-B2).

### Crystallization

*dr*UvrD^FL^ and *dr*UvrD^∆C^ were mixed with their respective DNAs at a 2:1 molar ratio in 50 mM Tris-HCl pH 8.0, 150 mM NaCl, 5 mM MgCl_2_, 5% glycerol, 0.1 mM EDTA, 1 mM DTT and 1 mM AMPPNP (Sigma) and concentrated to 8-10 mg/ml. Crystals were obtained using the hanging-drop vapor diffusion method at 20°C. *dr*UvrD^∆C^-For25/Rev25 form I crystals appeared very rapidly (<1 day) in 20% PEG 3350, 0.1 M Bis-Tris Propane pH 7.0 and 0.2 M Na-Nitrate, while form II crystals were obtained after at least one week in 22% PEG 3350, 0.1 M Bis-Tris Propane pH 7.5 and 0.1 M Na-Fluoride. High quality crystals of *dr*UvrD^FL^-For28/Rev28 suitable for data collection were obtained in 16-22% PEG 3350, 0.1 M Bis-Tris Propane pH 6.5-7.5 and 0.1-0.3 M Na-Nitrate after seeding. All crystals were cryoprotected by 20% glycerol and flash-frozen in liquid nitrogen. 5 mM AMPPNP was included in the cryoprotectants. 

### Data collection and Structure Determination

Diffraction data (Table 1) were collected at the European Synchrotron Radiation Facility (ESRF) in Grenoble, France and were processed with either *XDS* [21] or *iMosflm* [22]. The structure of *dr*UvrD^∆C^-For25/Rev25 form I was solved by molecular replacement using *Mr. Bump* [23] and the *gs*PcrA helicase as a search model (PDB entry 3PJR). After several rounds of substantial rebuilding of the protein chains in *Coot* [24], the DNA could be built and the AMPPNP molecules docked into the electron density. Subsequently, this model was used to solve the structures of *dr*UvrD^∆C^-For25/Rev25 form II and *dr*UvrD^FL^-For28/Rev28 by molecular replacement with *Phaser* [25]. The *dr*UvrD^∆C^-For25/Rev25 form I and form II models were refined with *Refmac* [26], while the *dr*UvrD^FL^-For28/Rev28 model, solved at lower resolution, was refined in *Phenix* [27] using *dr*UvrD^∆C^-For25/Rev25 form I as a reference model (Table 1). Fig.s of structures were prepared with *Pymol* [28] and the movie of the morph was created with *Chimera* [29].

**Table 1:** Crystallographic data collection and refinement statistics.

**Dataset**	***dr*UvrD^FL^**	***dr*UvrD^∆C^ form I**	***dr*UvrD^∆C^ form II**
**Data collection**			
Protein	*dr*UvrD^FL^	*dr*UvrD^∆C^	*dr*UvrD^∆C^
DNA	For28/Rev28	For25/Rev25	For25/Rev25
Nucleotide	AMPPNP	AMPPNP	AMPPNP
Space group	P2_1_	P2_1_2_1_2_1_	P2_1_
Cell dimensions *a*, *b*, *c* (Å) α,β,γ (°)	71.58, 390.58, 71.65 90.00, 106.00, 90.00	67.57, 67.45, 386.04 90.00, 90.00, 90.00	68.49, 89.79, 293.80 90.00, 89.97, 90.00
Beamline	ESRF ID14-4	ESRF ID14-2	ESRF ID23-1
Resolution (Å)	46.15 - 4.00 (4.22- 4.00)	47.40 - 2.55 (2.69 - 2.55)	47.63 - 3.00 (3.16 - 3.00)
*R*_merge_ (%)	10.6 (65.8)	7.1 (59.2)	6.4 (32.0)
<(I)/σ(I)>	10.1 (2.3)	20.4 (3.6)	6.5 (1.9)
Completeness (%)	99.6 (99.5)	100.0 (100.0)	89.0 (86.5)
**Refinement**			
N° of reflections (F > 0 σF )	30,051	56,088	59,507
R_fact_/R_free_ (%)	24.6/27.1	21.1/26.6	22.8/28.8
Mol/asu	4 chains UvrD 2 chains dsDNA	2 chains UvrD 1 chains dsDNA	4 chains UvrD 2 chain dsDNA
Ligands	4 AMP-PNP	2 AMP-PNP	2 AMP-PNP
Wilson B-factor	149.8	63.4	72.3
Average B-factor (Å^2^)			
Protein	203.0	63.9	102.2
DNA	256.8	163.9	132.0
AMPPNP	168.8	29.7	84.6
Solvent	N/A	38.5	84.3
Ramachandran			
Favoured (%)	93.8	89.1	89.0
Allowed (%)	6.1	10.6	10.7
Disallowed (%)	0.2	0.3	0.3
Rms deviations			
Bonds (Å)	0.006	0.017	0.012
Angles (°)	1.1	1.7	1.5
PDB ID	4c2t	4c2u	4c30

Values in parentheses are for highest resolution shell.

### ATPase activity

The rate of ATP hydrolysis by 100 nM *dr*UvrD^FL^ and *dr*UvrD^∆C^ in the presence of a 25mer polydT oligonucleotide was measured using the spectrophotometric method [30] at 25°C in 50 mM Tris–HCl pH 8.0, 100 mM NaCl, 0.1 mM EDTA, 1 mM DTT, 5 mM MgCl_2_ and 5% glycerol (buffer A). The K_ssDNA_ was determined by measuring the rate of ATP hydrolysis in the presence of 2 mM ATP as a function of increasing concentrations of ssDNA (0-10 µM). Kinetic parameters (V_max_, K_m_ and K_cat_) were determined by measuring the rate of ATP hydrolysis in the presence of an excess of ssDNA (10xK_ssDNA_) at various ATP concentrations (0-1 mM). The measurements were made in triplicate and the average ATPase rates were plotted and fitted to a hyperbola using *Origin*. 

### Helicase assay

Helicase activity of *dr*UvrD^FL^ was assayed in 10 mM Tris-HCl pH 8.0, 50 mM NaCl, 1% glycerol, 5 mM MgCl_2_ and 0.1 mg/mL BSA (buffer B). 80 µl reactions containing 20 nM DNA and 250 nM wild-type or mutant *dr*UvrD were incubated at 25°C. The duplexed DNA was either blunt or contained 15nt or 7nt ssDNA extensions at either the 3′- or 5′-ends. The reactions were initiated by addition of 2 mM ATP. At indicated time points, 10 µl samples of the reaction were quenched with 2.5 µl of a solution containing 0.8% SDS, 0.08% bromophenol blue, 24% glycerol, 80 mM EDTA and 20 µM unlabeled H1 oligonucleotide. The reactions were carried out in the absence and presence of 250 nM *dr*SSB. Reaction products were run on a 20 % polyacrylamide TBE gel and the DNA bands were visualized and quantified using a ChemiDoc MP imaging system and the Image Lab software (Bio-Rad). Initial reaction rates were estimated using GraphPad Prism6 and averaged data from three independent experiments were plotted in GraphPad Prism6 with standard deviations represented as vertical error bars.

### Streptavidin displacement assay

The translocase activity of *dr*UvrD was assayed using the streptavidin-displacement assay [31,32]. DNA oligonucleotides used in this assay consisted of dsDNA duplexes with a 25 nt ssDNA extension at either the 3′- or 5′-end and a biotin label in positions 49 and 2 respectively. The DNA-streptavidin complexes were formed by incubating the biotinylated dsDNA (0.2 µM) with streptavidin (3.2 µM, Sigma) in 10 mM Tris-HCl pH 8.0, 50 mM NaCl, 0.5 mM EDTA at 25°C for 30 min, before addition of 180 µM biotin. Displacement reactions of 80 µl containing 20 nM streptavidin-loaded DNA and 250 nM *dr*UvrD^FL^ were incubated in buffer B at 25°C. The reactions were initiated by addition of 2 mM ATP. At indicated time points, 10 µl samples of the reaction were quenched with 2.5 µl of a solution containing 0.48% SDS, 0.032% bromophenol blue, 20% glycerol, 160 mM EDTA and 20 µM unlabeled H1 oligonucleotide. The reactions were carried out in the absence and presence of 250 nM *dr*SSB. Reaction products were run on a 10 % polyacrylamide TBE gel and the DNA bands were visualized and quantified using a ChemiDoc MP imaging system and Image Lab software (Bio-Rad). Averaged data from three independent experiments were plotted in GraphPad Prism6 with standard deviations represented as vertical error bars.

### DNA Binding

Equilibrium DNA binding assays were performed on a Synergy H4 Hybrid Microplate reader (BioTek), fitted with polarization filters to measure fluorescence anisotropy. The binding assays were conducted in 384-well plates at room temperature in 80 µl reaction volumes in buffer A supplemented with 0.05% Tween 20, 0.1 mg/mL BSA and 1 mM AMPPNP. 0 to 8 µM wild-type and mutant *dr*UvrD were titrated into 2.5 nM fluorescently-labeled dsDNA containing 15nt ssDNA extensions at either the 3′- or 5′-end. Averaged data from three independent experiments were fitted to a standard binding equation (y=Bmax*x/(Kd+x)) assuming a single binding site [33] using GraphPad Prism6. The fits were very good, with R^2^ values all above 0.98.

## Results

### Crystal structures of drUvrD-DNA complexes

 A ternary complex containing 2 molecules of intact *dr*UvrD^FL^, a 21-mer DNA duplex with 7nt ssDNA extensions at each of its 3′-ends and the non-hydrolysable ATP analogue, AMPPNP, was crystallized in space group P2_1_ with four *dr*UvrD chains and two DNA duplexes per asymmetric unit (Figure 1B). These crystals diffracted X-rays to 4.0 Å (Table 1). Despite being present in the crystallized protein, residues 663-745 corresponding to the variable C-terminal region could not be traced in the electron density map, confirming that this region is particularly flexible [34]. Crystals containing the C-terminally truncated *dr*UvrD^∆C^ (Figure 1A), an 18-mer DNA duplex with 7nt ssDNA extensions at its 3′-ends (Figure 1B) and AMPPNP diffracted to higher resolution. This complex produced two crystal forms (I and II) diffracting respectively to 2.5 and 3.0 Å resolution (Table 1). Crystal form I appeared very rapidly (<1 day) and belonged to space group P2_1_2_1_2_1_ with two protein monomers and one DNA duplex per asymmetric unit, while crystal form II appeared after at least one week and belonged to space group P2_1_ with four molecules of protein and two DNA duplexes per asymmetric unit. In all structures, each *dr*UvrD monomer was bound to the ds-ssDNA junction at either end of the DNA duplex, thus forming an assembly of one DNA duplex with two UvrD monomers (Figure 1B). In the structures of *dr*UvrD^FL^ and *dr*UvrD^∆C^ form I, each protein monomer contains one bound AMPPNP molecule, whereas in *dr*UvrD^∆C^ form II each assembly is composed of a DNA duplex with an AMPPNP-bound UvrD on one end and an apo-UvrD on the other. 

 In all three structures, the quality of the electron density corresponding to the bound DNA varied considerably over the molecule. In contrast to the very well defined map of the ssDNA tails, the duplex regions were less clear and exhibited significantly higher B-factors than the adjacent protein atoms. In *dr*UvrD^∆C^ form I and *dr*UvrD^FL^, the nucleotides at the junction between the dsDNA and the ssDNA are poorly defined, indicating that this region is relatively flexible. 

**Figure 2:**
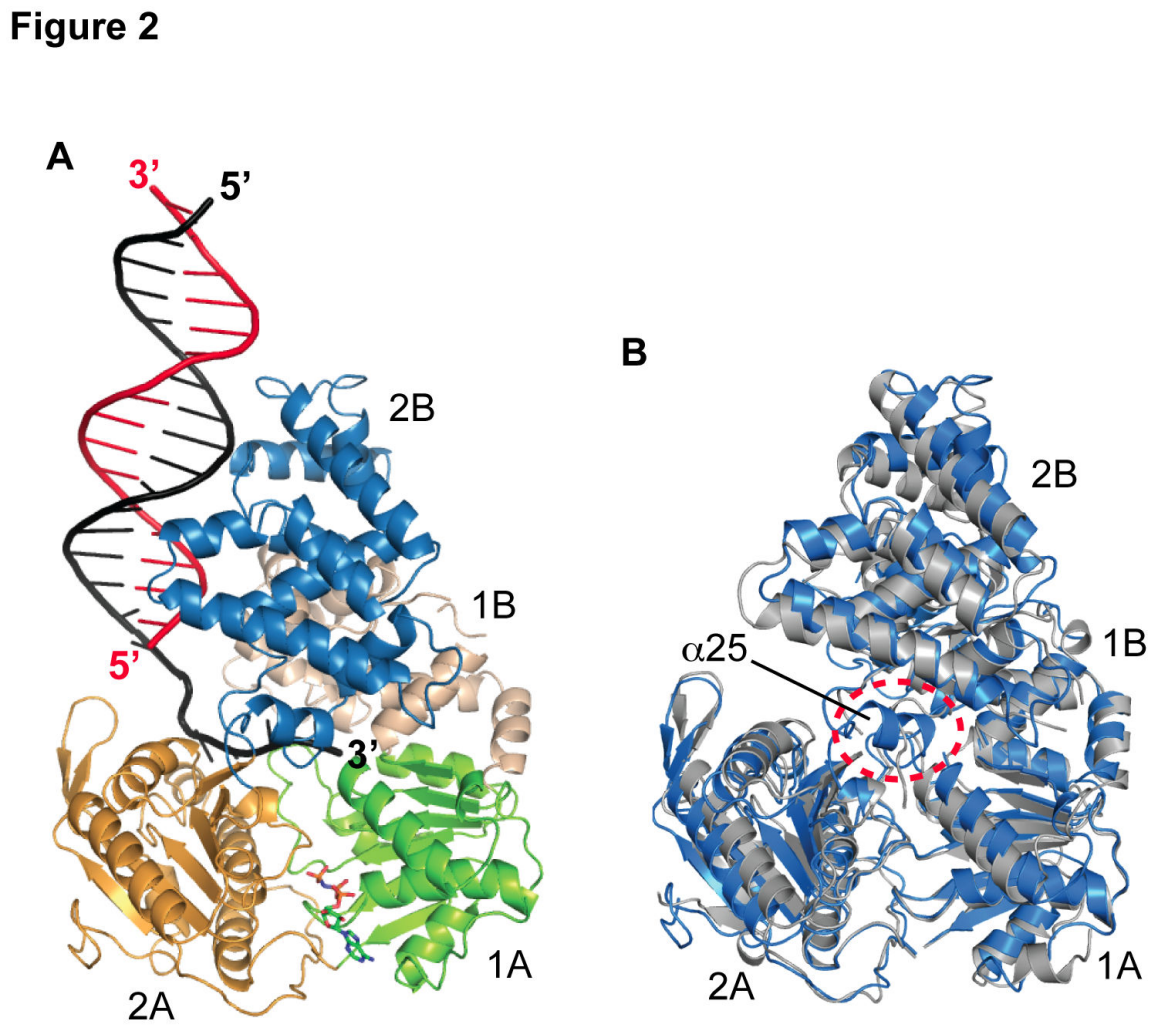
Structure of the *dr*UvrD helicase. A. Crystal structure of one monomer of *dr*UvrD^∆C^ bound to duplex DNA with a single-stranded extension at the 3′-end. The translocating strand is colored black and the complementary strand is colored red. The domains of *dr*UvrD^∆C^ are shown in ribbon and are colored green (1A), beige (1B), orange (a) and blue (2B). AMPPNP is shown in sticks. B. Overlay of nucleotide-bound *dr*UvrD (blue) and *ec*UvrD (grey) structures. The main structural difference is the linker between domains 2B and 2A that adopts a helical arrangement in *dr*UvrD (α25) as opposed to a flexible coil in *ec*UvrD.

 As in previous structures of UvrD-like helicases [17-19], *dr*UvrD crystallized as a monomer, and no putative dimer interfaces were detected between adjacent protein molecules in our three structures. *dr*UvrD displays 36% sequence identity with *E. coli* Rep (*ec*Rep) and UvrD (*ec*UvrD) helicases and 42% sequence identity with *G. stearothermophilus* PcrA (*gs*PcrA) helicase, all of which are members of the SF1A helicase family (Figure S1). The overall structures of the *dr*UvrD monomers are very similar to those observed in the closed conformation of *gs*PcrA-DNA and *ec*UvrD-DNA complexes [18,19] formed by domains 1A, 1B, 2A and 2B (Figure 2A and 2B). When present, AMPPNP is bound at the interface between domains 1A and 2A. ssDNA interacts with all four domains, a majority of contacts being with domain 2A, and interactions with dsDNA involve domains 1B, 2A and 2B (Figure 2A). 

 A close look at the residue conservation pattern (Figure S1) reveals that most of the non-conserved residues are found at domain interfaces. The buried surface areas and the nature of contacts at domain interfaces are indeed very different in *ec*- and *dr*UvrD (Table S2). In *dr*UvrD, the interface of domains 1B/2B is significantly smaller than in *ec*UvrD and the interfaces between domains 1A/1B and 1B/2B involve many more ionic interactions. Such differences may impart increased plasticity and flexibility to *dr*UvrD [35-37], but may also increase its sensitivity to stress-related changes in its local environment (e.g. pH, temperature, salt concentration).

### Large-scale conformational changes of drUvrD

**Figure 3:**
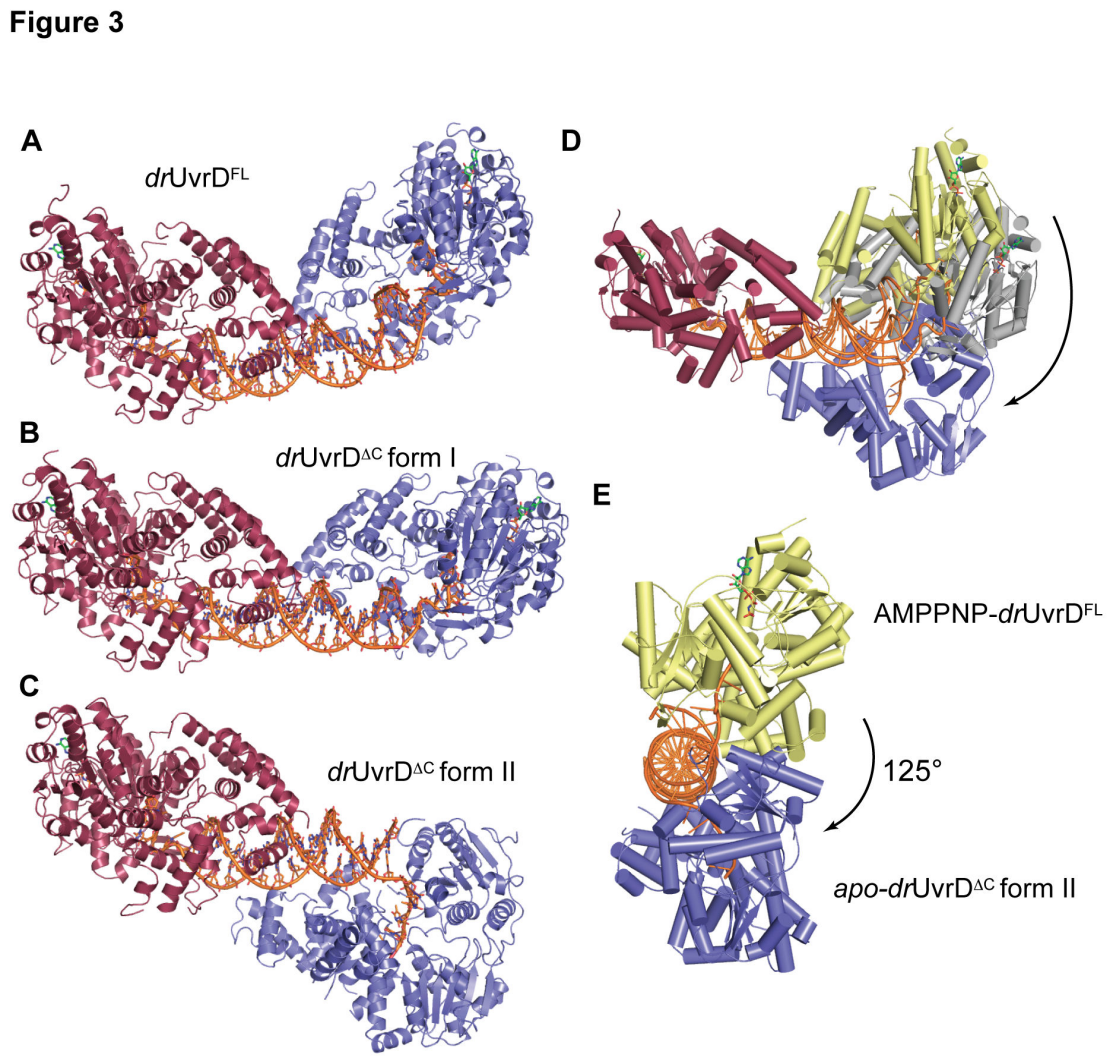
Crystal structures of *dr*UvrD-DNA complexes. A ribbon illustration of the AMPPNP-bound *dr*UvrD^FL^ is shown in A, the AMPPNP-bound *dr*UvrD^∆C^ form I is shown in B, the mixed AMPPNP-bound (red) and apo- (blue) *dr*UvrD^∆C^ form II is shown in C. The DNA and AMPPNP are shown in sticks. D-E. Large-scale conformational changes. D. Overlay of chains A (red) of *dr*UvrD^FL^, *dr*UvrD^∆C^ form I and apo-*dr*UvrD^∆C^ form II, illustrating the large spiral movement of chains B colored respectively yellow, grey and blue. The DNA is shown as an orange ribbon. E. As in (D) but viewed down the DNA axis, and for clarity *dr*UvrD^∆C^ form I has been removed.

 While the relative orientation of the components of the protein-DNA assembly observed in *dr*UvrD^∆C^ form II (Figure 3C) is similar to those observed in previous structures of SF1A helicases, our structures of *dr*UvrD^FL^ and *dr*UvrD^∆C^ form I provide us with two new snapshots of helicase-DNA complexes (Figure 3A and 3B). In both *dr*UvrD^FL^ and *dr*UvrD^∆C^ form I, the two *dr*UvrD protomers are located on the same side of the DNA duplex and induce a bend in the DNA (Table S3). In the case of *dr*UvrD^FL^, one of the two assemblies in the asymmetric unit forms a very bent assembly (25° bend in the DNA axis) where domains 2B of the two protomers come closer together (Figure 3A). The second protein-DNA assembly obtained in *dr*UvrD^FL^ crystals is more similar to the assembly obtained in *dr*UvrD^∆C^ form I. In these structures, the two *dr*UvrD protomers are still located on the same side of the DNA duplex (Figure 3B), but the DNA duplex is less bent (16° bend in the DNA axis; Table S3). In *dr*UvrD^∆C^, loss of one of the AMPPNP molecules leads to the formation of crystal form II in which the apo molecule has rotated 125° around the DNA with respect to the position of *dr*UvrD^FL^ or 105° with respect to crystal form I (Figure 3D and 3E), resulting in an assembly with one *dr*UvrD on either side of the DNA duplex (Figure 3C). In this assembly, the apo-*dr*UvrD^∆C^ molecule has twisted the 3′-ssDNA extension and maintains the ssDNA in a bent orientation with respect to the DNA duplex axis. As a result, the DNA duplex itself shows a reduced helical twist and reduced bending (Table S3). The accompanying movie (Movie S1) presents a morph of *dr*UvrD^∆C^ as it rotates around the duplexed DNA and undergoes this large spiral movement corresponding to the transition from crystal form I to crystal form II. 

### Local structural rearrangements of drUvrD

**Figure 4:**
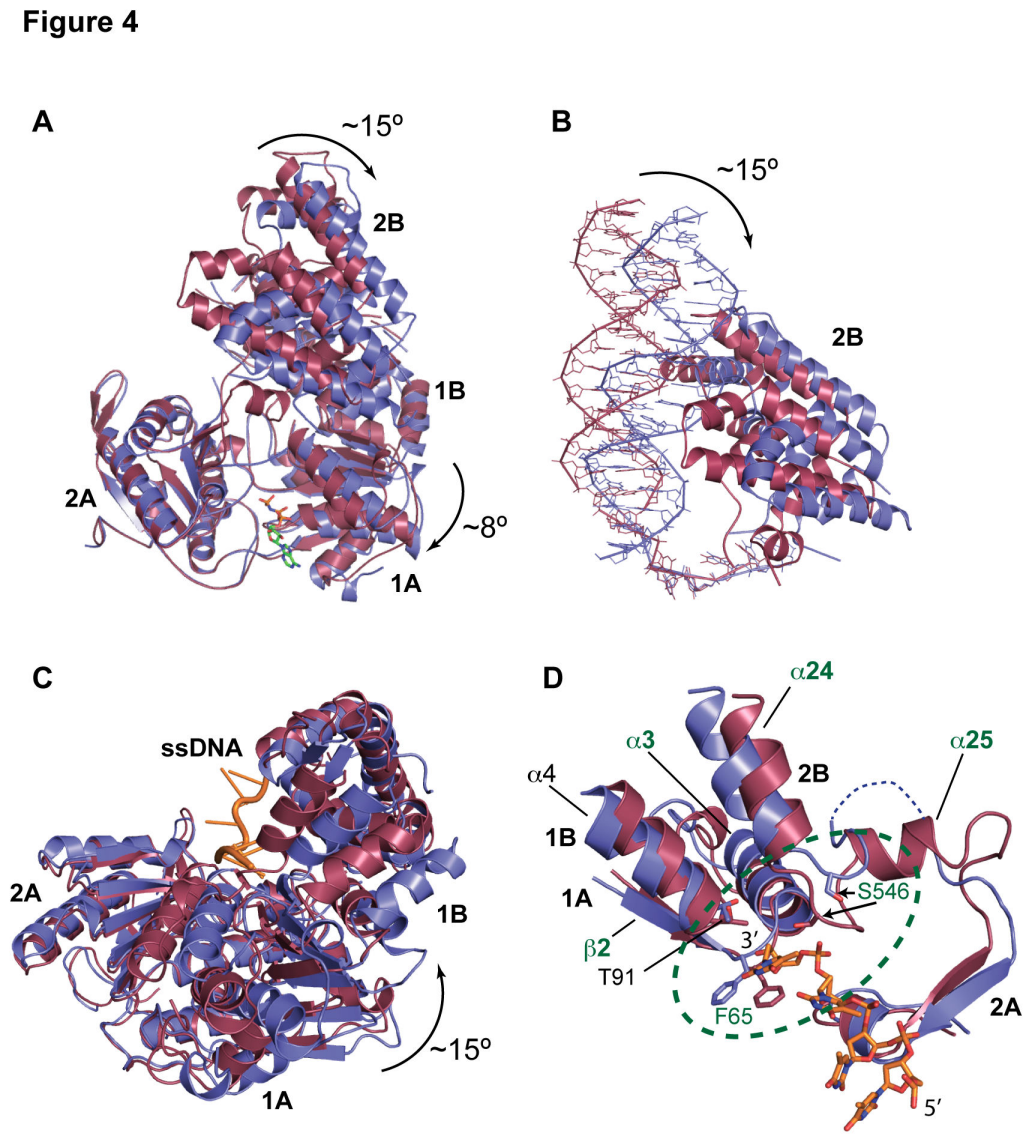
Conformational changes associated with ATP hydrolysis and nucleotide release. A-C. Domain movements. The AMPPNP-bound form is colored in red, while the apo-form is colored in blue. A. Upon ATP hydrolysis and nucleotide release, domain 2B along with the dsDNA rotates by ~15° and domain 1A and 1B by 8° relative to domain 2A. B. Close up view of the rotation of domain 2B and duplex DNA. C. Domains 1A and 1B undergo a 15° twist relative to domain 2A around the ssDNA axis (orange). D. Conformational changes occurring at the ssDNA gateway (circled in green). The linker between domains 2B and 2A adopts a short helix (α25) and loop in the AMPPNP-bound form and interacts tightly with the 3′-end of the ssDNA via Ser546, while it consists of an unstructured loop (dashed line) in the apo-form. In the AMPPNP form, the ssDNA gateway is more closed: the distance between the carboxyl oxygen of Phe65 (motif Ia) and the hydroxyl group of Ser546 is 4.5 Å in the AMPPNP-bound form versus 9.9 Å in the apo-form. The represented DNA corresponds to the AMPPNP bound form.

 The loss of the bound nucleotide also results in major structural rearrangements within the protein monomer (Figure 4). Loss of the nucleotide induces a ~15° rotation of domain 2B and an ~8° rotation of domains 1A and 1B relative to domain 2A in the plane formed by the ss- and dsDNA (Figure 4A and 4B). It also leads to a ~15° twist of domains 1A and 1B relative to domain 2A around the ssDNA axis (Figure 4C). Similar rotations have been observed previously in the structures of *gs*PcrA [18] and *ec*UvrD [19]. In *ec*UvrD, however, all three domains (1A, 1B and 2B) moved as a single unit as UvrD converted from a nucleotide-bound state to an apo-form and no significant changes in domain interactions were observed. In contrast, in *dr*UvrD domains 1A and 1B move independently of domains 2A and 2B and these movements lead to a number of structural rearrangements and the disruption of several salt bridges between domains 1A/1B and 1B/2B (Table S2). 

 Most conformational changes (within the protomer) associated with ATP hydrolysis involve a number of conserved sequence motifs (Figure S1) identified in *ec*UvrD [19] and described hereafter. In *ec*UvrD helix α24 from domain 2B was referred to as the gating helix and was proposed to regulate the exiting of ssDNA. In *dr*UvrD, this helix adopts a similar, closed conformation in both the nucleotide-bound and apo forms of *dr*UvrD (Figure 4D). This was also the case in the *gs*PcrA-DNA complexes [18] and in several of the nucleotide-bound and apo structures of *ec*UvrD [19]. In contrast, the linker that follows this helix and connects domain 2B to domain 2A (an unstructured loop in *ec*UvrD), adopts a different conformation depending on the nucleotide-bound state of *dr*UvrD. In AMPPNP-bound *dr*UvrD, the linker forms a loop (residues 544-548) and a small helix, α25 (Figures 2B and 4D), while in the nucleotide-free *dr*UvrD, this region is very flexible with part of the chain missing from the electron density maps, most likely to allow the ssDNA to exit the molecule. Helices α24 and α25 from domains 2B and 2A on one side and the conserved sequence motif Ia (β2 and α3) from domain 1A on the other side thus form an ssDNA gateway, which opens and closes like sliding doors (Figure 4D). In the AMPPNP bound form, the hydroxyl group of Ser546, on the linker between α24 and α25, is only 4.5 Å away from the carbonyl oxygen of Phe65 (motif Ia), thus blocking the ssDNA exit. In this form, the loop preceding α25 closes down on the 3′-end of the ssDNA and interacts directly via Ser546 with the phosphate of the terminal nucleotide. This conformation is stabilized by helix α25, which is missing in *ec*UvrD and is unstructured in apo-*dr*UvrD. Upon AMPPNP release, rotation of domains 1A, 1B and 2B opens the gateway; in apo-*dr*UvrD, the opening increases to nearly 10 Å to allow a single nucleotide to thread through. Additionally, in AMPPNP-bound *dr*UvrD, the ssDNA gateway is plugged by the tip of the β3- α4 loop (Thr91 from motif Ib) and this plug also moves out of the way in apo-*dr*UvrD to let the ssDNA through (Figure 4D).

### Mechanistic insight into DNA unwinding by drUvrD

**Figure 5:**
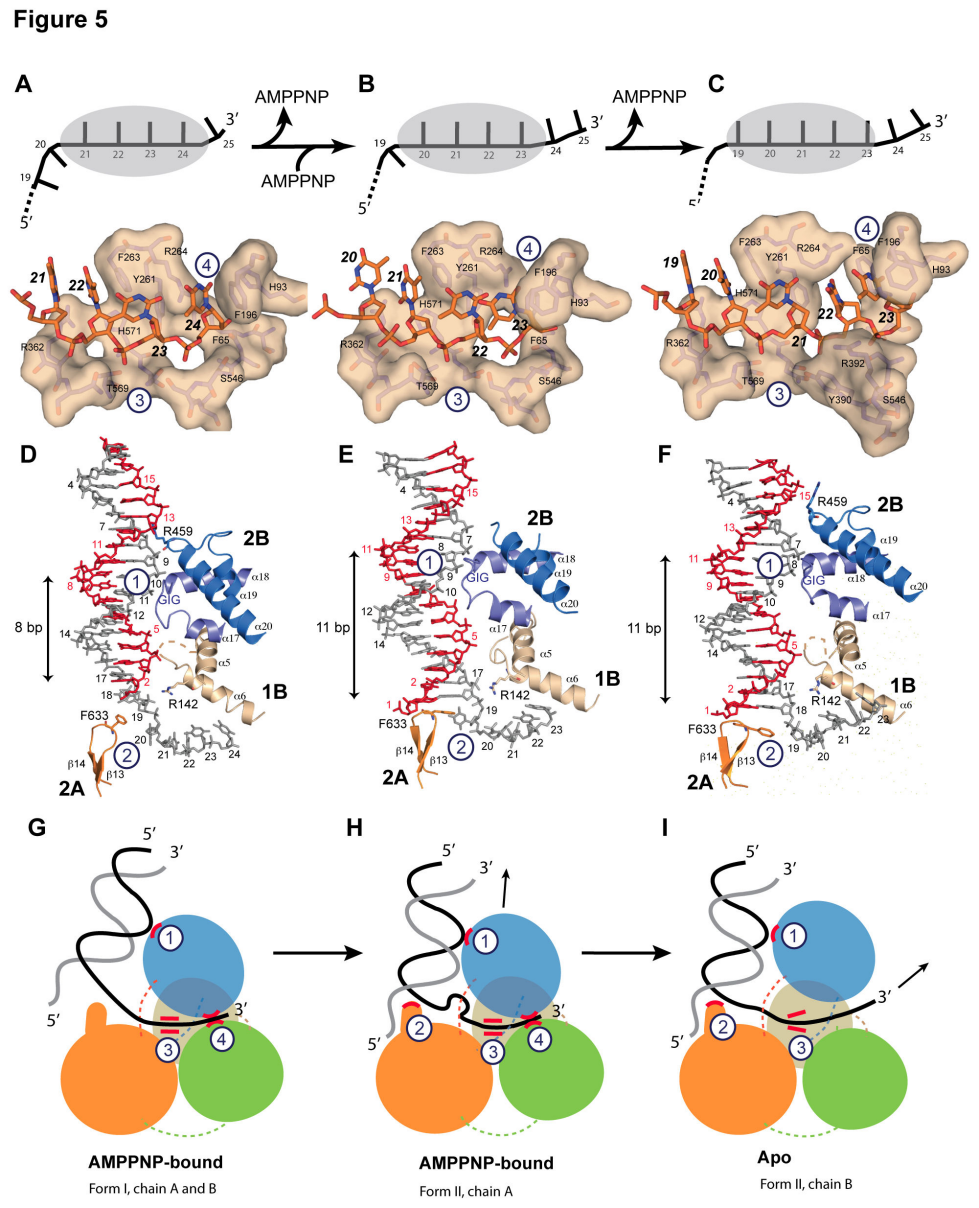
DNA binding of *dr*UvrD. Illustrations of *dr*UvrD binding to dsDNA with a 3′-ssDNA tail in form I (A,D and G), form II with AMPPNP bound (B, E and H) and in the apo-form of form II (C, F and I). A-C. Schematic diagrams (top) illustrating the translocation of form I (A), form II with AMPPNP bound (B) and the apo-form of form II (C) of *dr*UvrD^∆C^ along the ssDNA. The ssDNA nucleotides are illustrated as black bars and are numbered as in the crystal structures. The grey oval shape representing *dr*UvrD covers the nucleotides bound in the ssDNA binding pocket. Surface representations of the ssDNA binding pockets of these three forms of *dr*UvrD^∆C^ bound to ssDNA (orange sticks) are shown below. The important residues are labeled and the bases are numbered as in the schematic diagrams. D-F. Binding of *dr*UvrD^∆C^ to dsDNA in form I (D), form II with AMPPNP bound (E) and in the apo-form of form II (F). The dsDNA is illustrated in sticks with the translocated strand in grey. Domains of *dr*UvrD are colored as in Figure 2A. The helices belonging to the HLH motifs and the β-hairpin structure (orange) are shown and labeled according to the secondary structure succession (Figure S1). The positively charged residues in contact with dsDNA are illustrated in sticks and the GIG motif is indicated. The number of base-pairs formed between the ss-dsDNA junction and the contact point with the *dr*UvrD GIG motif is shown to the left of each panel. This number differs significantly between the two crystal forms. G-I. Schematic representation of *dr*UvrD's DNA binding in the different crystal structures as indicated below the models. The four protein-DNA contact points that are critical for the wrench-and-inchworm unwinding mechanism are indicated with circled numbers in all panels: HLH motifs interact with dsDNA (1), the β-hairpin motif with the ss-dsDNA junction (2), motif III with the ssDNA (3) and the ssDNA gateway with the exiting ssDNA (4). G. In AMPPNP bound Form I, contact points 1, 3 and 4 are tight. H. In AMPPNP bound Form II, *dr*UvrD's GIG motif (1) has slided along the DNA duplex and pushes the DNA junction against the β-hairpin motif (2), which now stacks tightly against the first base-pair. I. In the apo molecule of Form II, the ssDNA gateway (4) has opened and ssDNA exited the helicase. Domains of *dr*UvrD are colored as in Figure 2A.

 In *dr*UvrD^∆C^ form I, four nucleotides (nt21-24) are tightly bound in the ssDNA-binding pocket (Figure 5A). The terminal nucleotide (nt25) has already exited the helicase and is no longer visible in the electron density maps. Nucleotides 21 and 22 interact with Arg362 and Asn364 (motif IVa) and stack against Phe263 (motif III) that interferes with the regular stacking of the ssDNA bases and forces nucleotides 23 and 24 to adopt an orientation orthogonal to nucleotides 21 and 22. Nucleotide 24 is stabilized in this conformation by π-stacking of the base between Arg264 and Phe196 (motif Id) and of the deoxyribose ring against Phe65 (motif Ia). These residues are in turn stabilized by a series of stacking interactions involving notably Tyr261 (motif III) and His93 (motif Ib). In the AMPPNP-bound molecule of *dr*UvrD^∆C^ form II, nucleotides 20 to 23 are bound in the binding pocket (Figure 5B), indicating that *dr*UvrD has translocated along the ssDNA by one nucleotide compared to form I and as a result both nucleotides 24 and 25 have become untraceable. 

 As in *ec*UvrD, the apo-*dr*UvrD^∆C^ observed in crystal form II, reveals a fifth nucleotide in the ssDNA binding pocket (Figure 5C). Nucleotides 19 to 23 are now visible and sliding of *dr*UvrD along the DNA has positioned nucleotides 19 and 20 in the first two binding sites and nucleotides 21 and 22 in the subsequent sites. As a result, nucleotide 23 is now trapped on its way out. To allow the terminal nucleotide to exit, Phe196, Phe65 and His93 from motifs Ia, Ib and Id have moved out of the way and the α24-α25 linker that interacts via Ser546 with the terminal nucleotide in the AMPPNP–bound forms, has maintained its grip on the 3′-end of the ssDNA and pulled it through the opened gateway driven by the rotation of domain 2B (Figure 5C). Nucleotides 21 and 22 are now stabilized in their new binding sites by interactions with Tyr390 and Arg392 from motif IVb.

 Investigation of the dsDNA binding shows that it is also affected by the nucleotide-bound state of *dr*UvrD (Figure 5D-F). Interactions between *dr*UvrD and dsDNA involve four contact points: one helix-loop-helix (HLH) motif from domain 1B (α5-α6), two of the three HLH motifs from domain 2B (α17-α18 and α19-α20) and the β-hairpin motif (β13-β14) from domain 2A. In the AMPPNP-bound structures, three of these four sites are in contact with dsDNA; two of them are in common and the third differs between the two forms (Figure 5D and 5E). In both forms, Arg142 from the α5-α6 HLH motif interacts with the unpaired nt19 at the ss-dsDNA junction and the α17-α18 HLH motif containing the conserved GIG sequence (motif IVc, Figure S1) interacts extensively with nt9-12 in form I and nt7-10 in form II (Figure 5D and 5E). In form I, the third binding site involves Arg459 from the α19-α20 HLH motif, which interacts with the deoxyribose ring of nt13 (opposite strand) in the minor groove of the DNA duplex (Figure 5D), while in form II, Phe633 from the β-hairpin motif stacks against the first base-pair (nt1=nt18) of the duplex (Figure 5E). In the absence of nucleotide, however, only two of these contacts remain: the GIG sequence in the α17-α18 HLH motif interacts with nt7-10 and Phe633 from the β-hairpin motif stacks against the first base-pair (Figure 5F).

 Analysis of the ss- and dsDNA binding in the different structures (Figure 5) indicate how local conformational changes and domain rotations are transformed into *dr*UvrD's linear movement along the DNA via alternate loose and tight protein-DNA contact points, as proposed in the wrench-and-inchworm model for DNA unwinding. During ATP-binding-induced domain closing, binding to duplexed DNA through several HLH motifs (contact 1) and to ssDNA (contacts 3 and 4) are tight, while contact with the ss-dsDNA junction (contact 2) is loose (Figure 5D and 5G). UvrD then slides along the duplex away from the junction and thereby pushes the duplex DNA against the β-hairpin (contact 2). Phe633 located at the tip of the β-hairpin now stacks against the first base-pair (Figure 5E and 5H). Since the ssDNA gateway is closed at this stage, this movement creates a tension on the ss-dsDNA junction, which distorts the first nucleotide at the ss-dsDNA junction (nt19), thus forming a bulge (Figure 5E and 5H). This tension is then released during ADP and Pi release: domain rotations open *dr*UvrD’s ssDNA gateway (contact 4) to allow the ssDNA to exit the helicase. Contacts with the ssDNA (contact 3) remain tight throughout the process (Fig.s 5A-5C) in order to guide and tether the ssDNA through the gateway and straighten the bulged out nucleotide (Figure 5I). During this step, contacts with the DNA duplex are restricted to the GIG motif in α17-α18 HLH (contact 1 is looser) and the β-hairpin (contact 2) that is stacked against the first base-pair and is now in a position to act as a solid separation pin for subsequent unwinding of the duplex DNA (Figure 5F and 5I). 

### drUvrD is an active, DNA-stimulated ATPase and an ATP-dependent helicase

**Figure 6:**
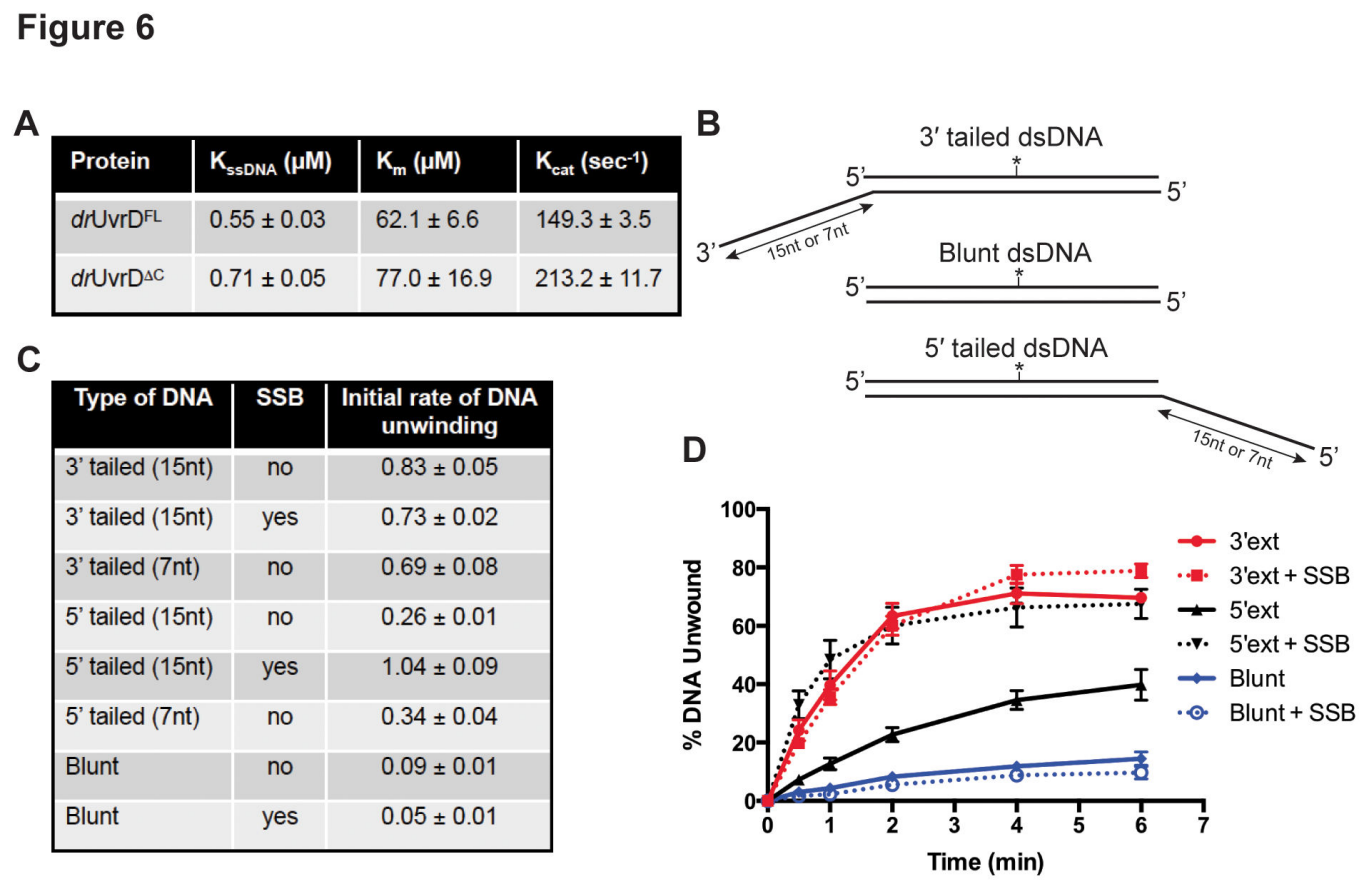
ATPase and helicase activity of *dr*UvrD. A. DNA-stimulated ATPase kinetic parameters of *dr*UvrD^FL^ and *dr*UvrD^∆C^. B. Structure of DNA oligonucleotides used for helicase assay of *dr*UvrD. The fluorescein label is represented as a star. C.-D. Helicase activity of *dr*UvrD^FL^ on DNA substrates shown in (B). C. Table summarising the initial rates of unwinding of duplexed DNA containing 15 or 7 nucleotide ssDNA extensions at either the 3′ or 5′ ends and of blunt duplexed DNA, as indicated, and in the absence and presence of *dr*SSB (250 nM). The rates are given in base-pairs per min per UvrD helicase unit (bp/min/UvrD). D. Time course of *dr*UvrD unwinding of duplexed DNA containing 15 nucleotide ssDNA extensions at either the 3′ (red) or 5′ (black)-ends and of blunt (blue) duplexed DNA in the absence (full line) and presence (dotted line) of *dr*SSB (250 nM). Standard deviations are shown as vertical bars.

 To better understand how a structurally and mechanistically conserved protein such as UvrD may be involved in diverse repair pathways in different species, we investigated *dr*UvrD’s catalytic activities *in vitro*. As other SF1A helicases, *dr*UvrD displays a clear DNA-stimulated ATPase activity (Figure 6A). Analysis of the ATPase data measured on *dr*UvrD^FL^ and *dr*UvrD^∆C^ allowed us to determine their apparent turnover rates (K_cat_) for ATP hydrolysis, along with their K_m_ for ATP and their K_ssDNA_ (corresponding to the concentration of ssDNA required for half-maximal ATPase rate) (Figure 6A). These values are in agreement with those measured for wild-type and a C-terminally truncated form of *ec*UvrD [34,38]. When compared to *dr*UvrD^FL^,*dr*UvrD^∆C^ exhibits a significantly higher turnover rate and reduced apparent affinities for both ATP and ssDNA, indicating that the C-terminal domain may be regulating the DNA binding and ATPase activities of *dr*UvrD. 

 We then examined the helicase activity of *dr*UvrD^FL^ on 3′-tailed, 5′-tailed and blunt dsDNA (Figure 6B). Our data reveals that *dr*UvrD unwinds all three of these substrates in an ATP-dependent manner to varying extents and, as expected for a member of the SF1A helicase family, unwinds preferentially 3′-tailed dsDNA (Figure 6C and D and Figure S2). The length of the ssDNA overhangs did not significantly affect the helicase activity of *dr*UvrD, since very similar initial rates of unwinding were observed for 15nt and 7nt overhangs (Figure 6C). Although *dr*UvrD shows a preference for unwinding 3′-tailed dsDNA, *dr*UvrD also melts 5′-tailed DNA at a 3-fold lower rate and blunt dsDNA at a 9-fold lower rate. In the presence of *dr*SSB (added at a 12.5-fold excess with respect to the DNA), the helicase activity on 3′-tailed and blunt dsDNA was unaffected, whereas *dr*UvrD’s activity on 5′-tailed dsDNA was strongly stimulated to a rate similar to that observed on 3′-tailed dsDNA (Figure 6C and D and Figure S2). In these conditions, *dr*UvrD could unwind duplexed DNA with the same efficiency in both directions. SSB has previously been reported to directly stimulate the helicase activity of several other helicases [39,40]. Interestingly, we also observed helicase activity on 3′-tailed dsDNA containing a fluorescein-conjugated thymine within its ssDNA extension (Figure S2) and succeeded in crystallizing *dr*UvrD^∆C^ in complex with such a DNA. Data to 3.0Å resolution were collected on crystals with modified DNA at position 21 and extra electron density could be seen close to the C7 group of thymine 21 (Figure S3). Several DNA helicases have previously been shown to unwind lesion-containing DNA [41]. *ec*UvrD was previously shown to efficiently unwind thymine glycol containing DNA [42] and *E. coli* Rep can efficiently unwind a DNA substrate harboring a polyglycol linkage in the ssDNA extension [43]. These findings suggest that although UvrD helicases bind tightly to ssDNA, they are sufficiently flexible to allow bases with bulky modifications through their ssDNA gateway. 

### drUvrD translocates on ssDNA in the 3′-5′ direction only

**Figure 7:**
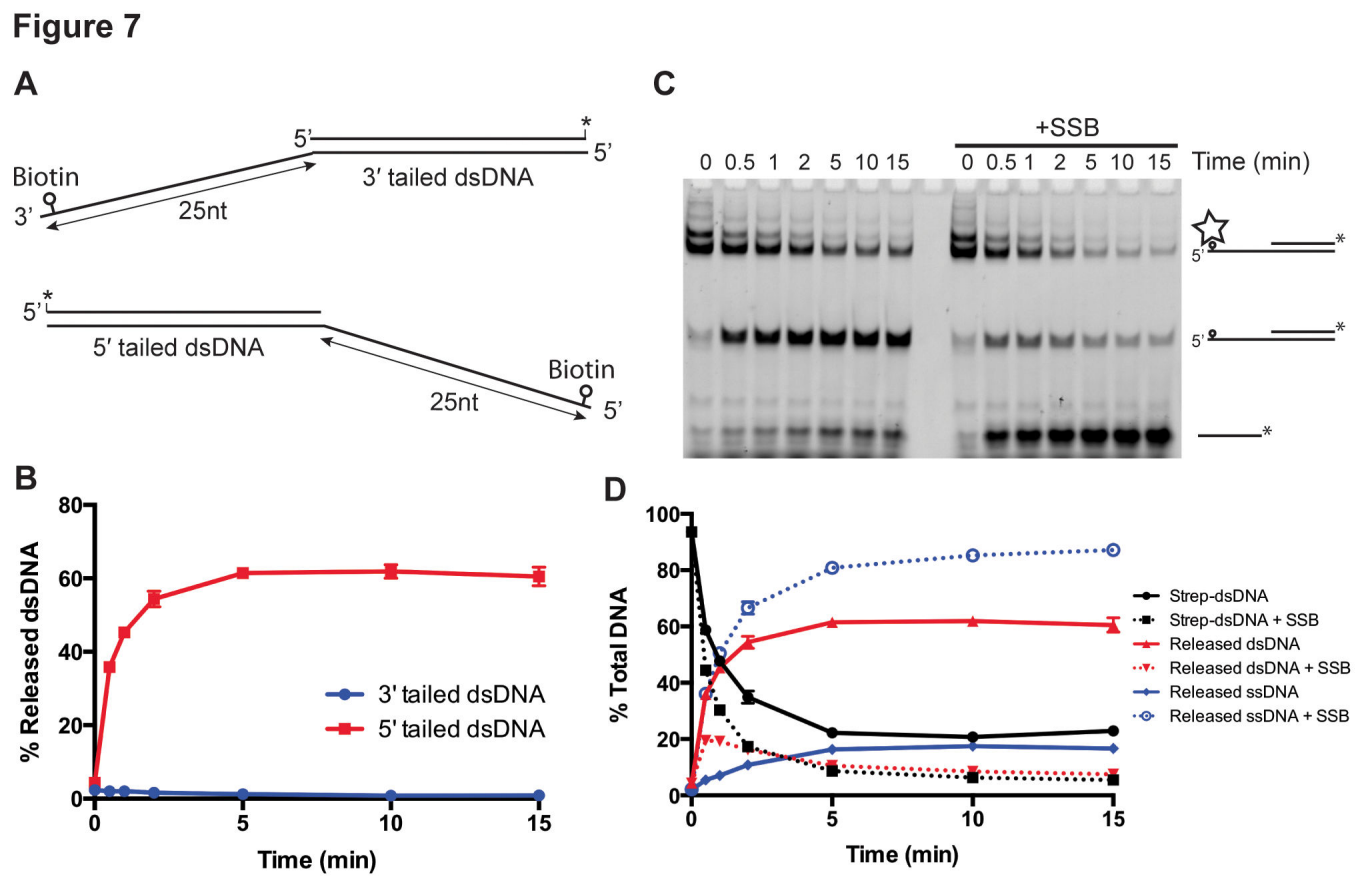
ssDNA translocase activity of *dr*UvrD. Translocase activity of *dr*UvrD was assayed using the streptavidin-displacement assay. A. Structure of DNA oligonucleotides used for *dr*UvrD translocase assay measuring streptavidin displacement from biotinylated DNA substrates. The fluorescein label is represented as a star and the biotin label as a circle. B. Time course of *dr*UvrD (250 nM) catalyzed streptavidin displacement from the 3′- (blue) and 5′- (red) ssDNA extensions of DNA oligonucleotides shown in (A). The fraction of released dsDNA (no longer bound to streptavidin) was quantified and plotted as a function of time. C. Translocase activity of *dr*UvrD (250 nM) on 5' tailed dsDNA (20 nM) as a function of time in the absence (left) and the presence (right) of *dr*SSB (250 nM). The reaction products were analyzed on a 10 % polyacrylamide TBE gel. Bands correspond to the fluorescein labeled reaction products: streptavidin-bound dsDNA (upper bands, corresponding to several biotin labeled oligonucleotides bound to streptavidin), released dsDNA (middle band) and unwound ssDNA (lower band). D. The bands shown in (C), resulting from the time course of streptavidin displacement from 5′- tailed dsDNA, were quantified and the fraction of streptavidin-bound (black), released dsDNA (red) and unwound ssDNA (blue) were plotted as a function of time for reactions carried out in the absence (full lines) and presence (dotted lines) of *dr*SSB (250 nM). Standard deviations are shown as vertical bars.

 We then investigated *dr*UvrD’s ability to translocate on ssDNA using a streptavidin-displacement assay (Figure 7). We found that *dr*UvrD could efficiently release streptavidin bound to biotin located at the end of 5′-tails in an ATP-dependent manner, but failed to displace streptavidin bound to biotin at the end of 3′-tails (Figure 7B and Figure S4), indicating that *dr*UvrD translocates along ssDNA in the 3′-5′ direction. Here again, as in the helicase assays, addition of *dr*SSB to the reaction mix did not affect translocation along 3′-tails, but significantly reduced the translocase activity on 5′-tails (Figure 7C). Interestingly, the addition of *dr*SSB leads to both a reduction in the amount of streptavidin-free 5′-tailed dsDNA (middle band on the gel, corresponding to the product of the translocase activity) and a major increase in the amount of released ssDNA (lower band on the gel, corresponding to the product of the helicase activity). *dr*SSB therefore modulates the activity of *dr*UvrD on 5′-tailed dsDNA. In the absence of *dr*SSB, *dr*UvrD preferentially translocates along the ssDNA in the 3′-5′ direction, whereas in the presence of *dr*SSB that most likely binds to the 5′ ssDNA extension, *dr*UvrD preferentially unwinds 5′-tailed dsDNA in the 5′-3′ direction through an as yet unidentified mechanism.

### Mutagenesis study of drUvrD’s DNA binding ability and helicase activity

**Figure 8:**
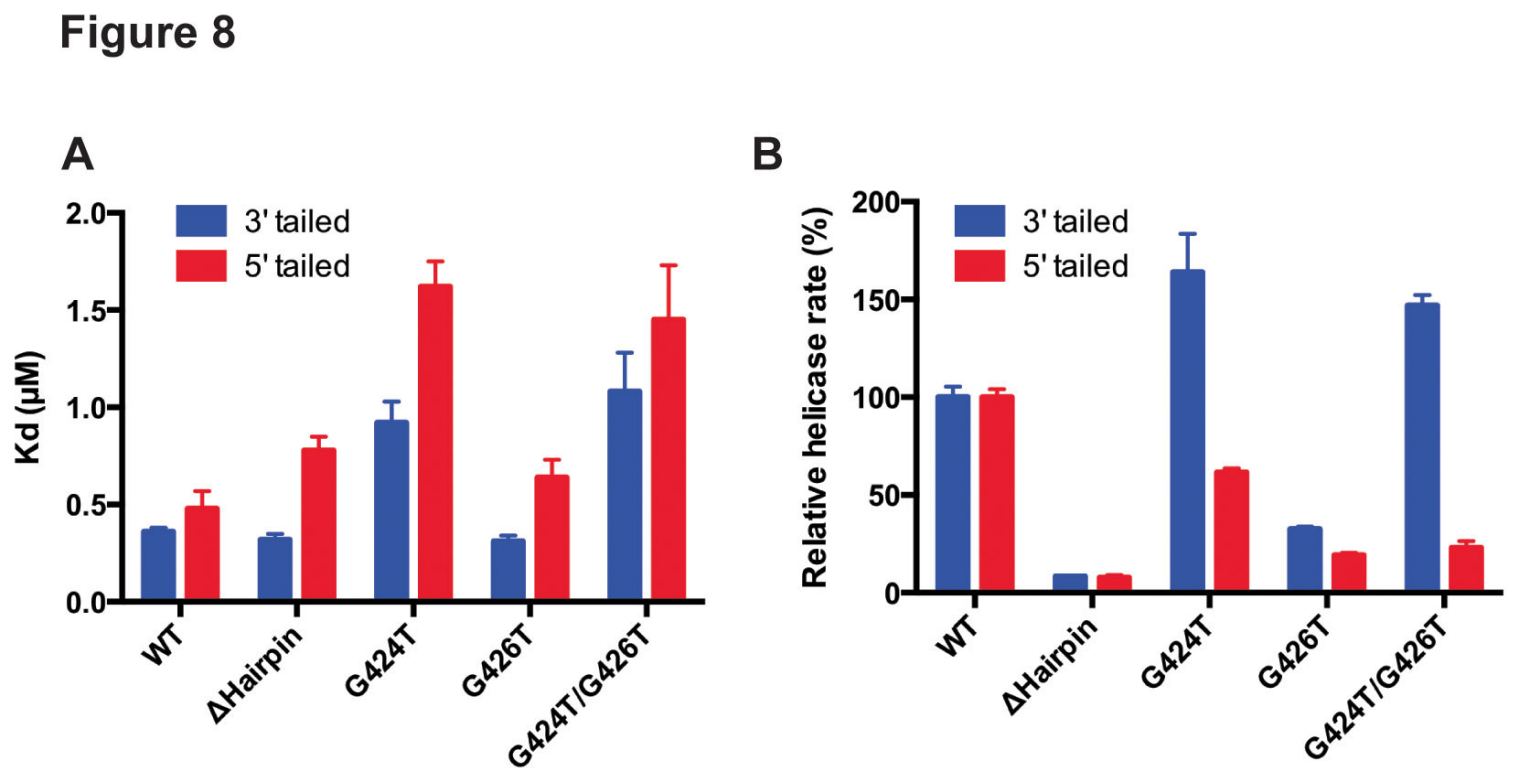
DNA binding ability and helicase activity of drUvrD mutants. Comparison of DNA binding ability and helicase activity of wild type (WT) and *dr*UvrD mutants: β-hairpin deletion mutant (ΔHairpin), and mutants of the GIG motif from domain 2B involved in dsDNA binding (G424T, G426T and double mutant G424T/G426T). A. DNA binding affinities (K_d_ values) of WT and mutant *dr*UvrD for either 3'-tailed (blue) or 5'-tailed (red) dsDNA determined by fluorescence anisotropy measurements. B. Helicase activity of WT and mutant *dr*UvrD (250 nM) on 3'-tailed (blue) or 5'-tailed (red) dsDNA (20 nM). Initial reaction rates were determined from reaction time courses and were normalized with respect to the activity of WT *dr*UvrD. Standard deviations are shown as vertical bars.

 Directionality of SF1 helicases is believed to be determined by preferential binding to either a 3′- or a 5′-ssDNA overhang, which acts as the entry point for the helicase [4]. We carried out fluorescence anisotropy measurements to evaluate the affinity of *dr*UvrD for either 3′- or 5′-tailed dsDNA (Figure 8A and Figure S5). *dr*UvrD binds to both of these substrates with similar affinity. The binding of *dr*UvrD to 3’-tailed dsDNA is slightly stronger than to 5′-tailed dsDNA (K_d_ for 3′-tailed dsDNA: 0.36 µM, K_d_ for 5′-tailed dsDNA: 0.48 µM). These values are also very close to the estimated affinity of *dr*UvrD^FL^ for ssDNA derived from our ATPase data (K_ssDNA_: 0.55 µM; Figure 6A). We mutated residues identified in *ec*UvrD [19] as being essential for DNA unwinding using the wrench-and-inchworm mechanism (Gly424 and Gly426 from the GIG motif and the β-hairpin) and tested the DNA binding and helicase activities of these *dr*UvrD mutants on 3′- or 5′-tailed dsDNA (Figure 8). Mutating Gly426 to threonine (G426T) did not significantly affect the binding of *dr*UvrD to 3′- and 5′-tailed dsDNA, whereas mutating Gly424 to threonine (G424T) alone or together with the G426T mutation significantly impaired the binding of *dr*UvrD to both 3′- and 5′-tailed dsDNA (K_d_ values increased by 3-4 fold; Figure 8A and Figure S5). Deletion of the β-hairpin structure, which is known to act as a separation pin and is essential for *ec*UvrD’s helicase activity [19], did not affect *dr*UvrD’s binding to 3′-tailed dsDNA and led to a slightly reduced affinity for 5′-tailed dsDNA (Figure 8A and Figure S5). These mutations, however, had a much more dramatic effect on the helicase activities of *dr*UvrD (Figure 8B). Deletion of the β-hairpin dramatically reduced DNA unwinding of both 5′- and 3′-tailed dsDNA and this was also the case for the G426T mutant. In contrast, *dr*UvrD-G424T mutants (single and double) showed a highly stimulated helicase activity on 3′-tailed dsDNA, as has previously been observed for *ec*UvrD [19], and a reduced activity on 5′-tailed dsDNA. These results suggest that *dr*UvrD’s 5′-3′ helicase activity relies on both a functional separation pin and tight binding to duplexed DNA via its GIG motif, whereas its 3′-5′ activity only requires the β-hairpin structure. 

## Discussion

 Because of the helical nature of nucleic acids, helicases are expected to translocate along DNA in a spiral movement. For the first time, our structures trap this large-scale spiral movement and reveal how the combination of rotational and translational movements, associated with the positioning of the helicase at an angle relative to the dsDNA axis produce a spiral trajectory along the DNA duplex. In addition, our two higher resolution structures of *dr*UvrD^∆C^ provide new insight into the detailed mechanisms underlying ATP-dependent DNA unwinding. Although the details of the protein-DNA contacts are not strictly identical in the structures of *dr*UvrD, *ec*UvrD [19] and *gs*PcrA [18], taken together, our observations suggest that the molecular mechanisms underlying this complex process are highly conserved within the SF1A helicase superfamily and support the tightly regulated wrench-and-inchworm model. The main differences we observe concern the gating mechanism regulating the exiting of the ssDNA. As in previous crystallographic studies of SF1 helicases [17-19], our crystal structures reveal no direct protein-protein contacts between neighboring UvrD monomers, even in the crystal structure of the intact *dr*UvrD^FL^, in which the duplexed DNA is significantly bent, bringing the two UvrD monomers close to each other (Figure 3A). 

Despite being structurally and mechanistically conserved with *ec*UvrD and *gs*PcrA, to our surprise, our biochemical assays revealed that *dr*UvrD differs from its homologues in a number of ways. We found that, unlike *ec*UvrD, *dr*UvrD could efficiently unwind dsDNA with only short (7nt) ssDNA overhangs. It is clear from our crystal structures that only a single *dr*UvrD monomer can bind to such a short ssDNA tail. Although our data do not allow us to determine the active oligomeric state of *dr*UvrD, our findings suggest that its helicase activity only requires that one UvrD monomer be loaded on the ssDNA tail. Our data also revealed that *dr*UvrD can efficiently translocate along ssDNA with a biased 3′-5′ directionality as observed previously for *ec*UvrD [44-46], but in contrast can melt both 3′- and 5′-tailed DNA duplexes. This is consistent with our finding that *dr*UvrD binds to both types of DNA. *dr*UvrD also displayed a weak helicase activity on blunt DNA. Most members of the SF1A family show a clear 3′-5′ polarity [38,47,48]; there are, however, several examples of enzymes including the PcrA helicase, notably in gram-positive bacteria, that show bipolar helicase activity [49-54]. Several UvrD homologues are also known to act on blunt or nicked DNA [34,47,49,54,55]. Our findings now provide further evidence that SF1A helicases vary both in terms of substrate specificity and helicase polarity. 

**Figure 9:**
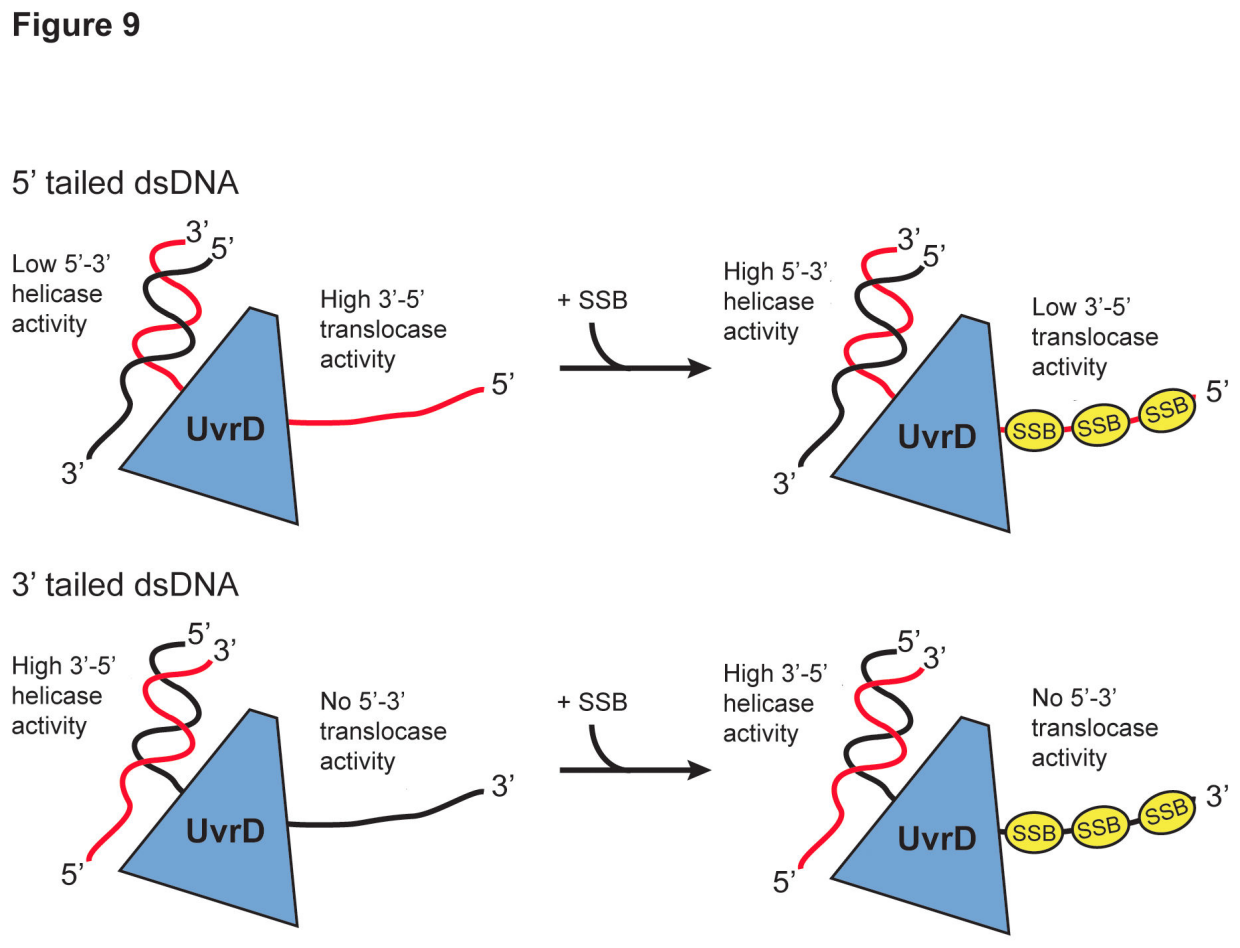
Model of DNA duplex unwinding and ssDNA translocation by *dr*UvrD. Models of *dr*UvrD DNA unwinding and ssDNA translocase activity on 5' tailed dsDNA (top) and 3' tailed dsDNA (bottom) in the absence (left) and presence (right) of *dr*SSB. Using 5' tailed dsDNA, in the absence of *dr*SSB *dr*UvrD has low 5'-3' helicase activity and high 3'-5' translocase activity while, in the presence of *dr*SSB, *dr*UvrD has high helicase activity and low translocase activity. Using 3'-tailed dsDNA, *dr*UvrD has high 3'-5' helicase activity and no 5'-3' translocase activity, regardless of the absence or presence of *dr*SSB.

Interestingly, our experiments carried out in the presence of *dr*SSB, which is known to coat and protect nascent ssDNA *in vivo*, reveal that SSB plays an important role in modulating the balance between helicase and translocase activity on 5′-tailed dsDNA (Figure 9). The presence of SSB strongly favors the helicase versus translocase activity of *dr*UvrD on such a substrate. This effect could be due to a direct regulation of *dr*UvrD’s activity by SSB or more likely to a steric effect of SSB binding to the ssDNA extension. In contrast, SSB does not appear to have any effect on *dr*UvrD’s activity on 3′-tailed dsDNA (Figure 9).

Our mutagenesis, DNA binding and helicase activity data indicate that regardless of the DNA substrate, the GIG motif of *dr*UvrD is critical for DNA binding and the β-hairpin structure is essential for DNA unwinding of both 5′- and 3′-tailed DNA substrates. The GIG motif and the β-hairpin separation pin are two essential features of the wrench-and-inchworm mode of unwinding and appear to be involved in both the 3′-5′ and on 5′-3′ helicase activities of *dr*UvrD. However, we also observe that mutating Gly424 from the GIG motif has a very contrasted effect on 3′-5′ and on 5′-3′ helicase activity (stimulated 3′-5′ activity and reduced 5′-3′ activity), indicating that the GIG motif from domain 2B may be regulating these two processes differently. A number of *ec*UvrD mutants, including GIG mutants, are known to display reduced DNA binding and yet robust 3′-5′ helicase activity as observed for *dr*UvrD [19,56]. This has been proposed to result from an alternative mode of unwinding, known as strand-displacement, in which movement of ssDNA is deregulated due to reduced contacts with dsDNA. This mechanism has been reported, notably in the absence of domain 2B and duplex DNA binding [19,57]. In the case of *dr*UvrD, impaired DNA binding may cause *dr*UvrD to switch from the controlled wrench-and-inchworm to an unregulated strand-displacement mode of unwinding on 3′-tailed DNA. In such a mode, the rotational movement of domain 2B is no longer coupled to ATP binding and hydrolysis and as a result domain 2B is no longer needed and may adopt a more open conformation, as observed in the DNA-free structures of *gs*PcrA and *ec*UvrD [16,58]. 

The targeting and involvement of helicases in distinct cellular repair processes thus appears to be achieved by their abilities to bind and unwind specific structures corresponding to intermediates of these processes. For example, the 5′-3′ unwinding activity of *Staphylococcus aureus* PcrA helicase is greatly stimulated in the presence of specific DNA structures [50]. *dr*UvrD’s ability to unwind 5′- and 3′-tailed DNA duplexes and containing modified bases within the translocating strand may reflect its implication in diverse DNA repair pathways *in vivo*. In *E. coli*, recombinational repair has been proposed to involve the 3′-5′ helicases RecQ and Helicase IV and the 5′-3′ helicase RecD, while *D. radiodurans* cells missing these genes show wild-type radioresistance and DNA repair capacity [7,59]. In contrast, inactivation of *dr*UvrD leads to a significant increase in the sensitivity of cells to γ-irradiation [7]. This phenotype is further enhanced in cells in which both *uvrD* and *recD2* genes have been disrupted, suggesting that the 5′-3′ helicase, *dr*RecD2, may in part back-up *dr*UvrD’s function. While further studies will be needed to decipher the detailed molecular mechanisms that regulate the helicase activities of *dr*UvrD, these observations suggest that *in vivo* both helicase activities of *dr*UvrD are needed. *dr*UvrD may switch between its translocase and helicase activities in response to external stresses, changes in its environment, or, as suggested by our experiments in the presence of SSB, upon interactions with pathway-specific protein partners such as SSB, MutL or UvrAB [9,55,60].

## Supporting Information

Table S1 **Sequences of DNA oligonucleotides used in this study.**
(DOCX)

Table S2 **Nature of contacts between the various domains of nucleotide-bound *ec*- and *dr*UvrD.**
(DOCX)

Table S3 **Summary of the helical parameters of the DNA duplexes bound to *dr*UvrD compared to ideal B-form DNA.**
(DOCX)

Movie S1 **DNA unwinding by *dr*UvrD.** The movie presents a morph between *dr*UvrD^∆C^ form I and *dr*UvrD^∆C^ form II. Molecule A (red) is in an AMPPNP-bound form in both cases, while molecule B (blue) converts from an AMPPNP-bound form (I) to an apo-form (II).
(MOV)

Figure S1 **Sequence alignment of *D. radiodurans* UvrD, *E. coli* UvrD, *E. coli* Rep and *G. stearothermophilus* PcrA helicases.** The secondary structure of *dr*UvrD is shown above the alignment and the domains are illustrated as colored lines below the alignment. The domains are colored as in Figure 1. The conserved helicase motifs are numbered and marked with yellow boxes.
(TIF)

Figure S2 **Helicase activity on 3′-, 5′-tailed and blunt dsDNA.** A. *dr*UvrD (250nM) unwinding of 3′-tailed 25 base-pair dsDNA (20nM) with either 15nt- or 7nt ssDNA extensions in the absence and presence of SSB (250nM). B. *dr*UvrD (250nM) unwinding of blunt 25 base-pair dsDNA (20nM) in the absence and presence of SSB (250nM). C. *dr*UvrD (250nM) unwinding of 5′-tailed 25 base-pair dsDNA (20nM) with either 15nt- or 7nt ssDNA extensions in the absence and presence of SSB (250nM). A-C. Reactions were stopped at the following time points: 0, 30sec, 1min, 2min, 4min and 6min, prior to separation on 20% TBE gels. The fluorescein label is illustrated as a star in the schematic representation of the DNA.
(TIF)

Figure S3 **Binding of fluorescein-labeled DNA to *dr*UvrD^∆C^.** The DNA oligonucleotides contain a fluorescein-conjugated thymine at position 21 within the ssDNA extension. The 2Fo-Fc electron density map (blue) is contoured at 1σ, while the Fo-Fc difference density map (green) is contoured at 2.5σ. The ssDNA is illustrated in sticks.
(TIF)

Figure S4 **Translocase activity on 3′- and 5′-tailed dsDNA.** *dr*UvrD (250nM) translocation activity on streptavidin bound 5′- and 3′-tailed 25 base-pair dsDNA (20nM) with 25nt ssDNA extensions. Reactions were stopped at the following time points: 0, 30sec, 1min, 2min, 5min, 10 min and 15min, prior to separation on 10% TBE gels. The fluorescein and the biotin labels are illustrated respectively as a star and an open circle in the schematic representation of the DNA. The upper bands correspond to streptavidin-bound dsDNA substrate, the middle-band to the released dsDNA (translocase product) and the lower band corresponds to the product of the helicase activity of UvrD, i.e. ssDNA. Biotinylated dsDNA without streptavidin was loaded in the first well.
(TIF)

Figure S5 **DNA binding to 3′- and 5′-tailed dsDNA.** Binding of wild-type (WT) and mutant drUvrD to 3′- (A) and 5′-tailed dsDNA (B) was measured by fluorescence anisotropy. The anisotropy measured for the DNA alone was subtracted from all other values and the change in anisotropy (ΔA) is plotted as a function of UvrD concentration (µM). The averaged data points were fitted to a standard binding equation assuming a single binding site using GraphPad Prism6. Standard deviations are shown as vertical bars.
(TIFF)

## References

[1] Tuteja N, Tuteja R (2004) Unraveling DNA helicases. Motif, structure, mechanism and function. Eur J Biochem 271: 1849-1863. doi:10.1111/j.1432-1033.2004.04094.x. PubMed: 15128295. 15128295

[2] Tuteja N, Tuteja R (2004) Prokaryotic and eukaryotic DNA helicases. Essential molecular motor proteins for cellular machinery. Eur J Biochem 271: 1835-1848. doi:10.1111/j.1432-1033.2004.04093.x. PubMed: 15128294. 15128294 PMC7164108

[3] Gorbalenya AE, Koonin EV (1993) Helicases: amino acid sequence comparisons and structure-function relationships. Curr Opin Struct Biol 3: 419-429. doi:10.1016/S0959-440X(05)80116-2.

[4] Singleton MR, Dillingham MS, Wigley DB (2007) Structure and mechanism of helicases and nucleic acid translocases. Annu Rev Biochem 76: 23-50. doi:10.1146/annurev.biochem.76.052305.115300. PubMed: 17506634. 17506634

[5] Bruand C, Ehrlich SD (2000) UvrD-dependent replication of rolling-circle plasmids in Escherichia coli. Mol Microbiol 35: 204-210. doi:10.1046/j.1365-2958.2000.01700.x. PubMed: 10632890. 10632890

[6] Arthur HM, Lloyd RG (1980) Hyper-recombination in uvrD mutants of Escherichia coli K-12. Mol Gen Genet 180: 185-191. doi:10.1007/BF00267368. PubMed: 7003307. 7003307

[7] Bentchikou E, Servant P, Coste G, Sommer S (2010) A major role of the RecFOR pathway in DNA double-strand-break repair through ESDSA in Deinococcus radiodurans. PLOS Genet 6: e1000774. 20090937 10.1371/journal.pgen.1000774 PMC2806897

[8] Veaute X, Delmas S, Selva M, Jeusset J, Le Cam E et al. (2005) UvrD helicase, unlike Rep helicase, dismantles RecA nucleoprotein filaments in Escherichia coli. EMBO J 24: 180-189. doi:10.1038/sj.emboj.7600485. PubMed: 15565170. 15565170 PMC544901

[9] Matson SW, Robertson AB (2006) The UvrD helicase and its modulation by the mismatch repair protein MutL. Nucleic Acids Res 34: 4089-4097. doi:10.1093/nar/gkl450. PubMed: 16935885. 16935885 PMC1616947

[10] Caron PR, Kushner SR, Grossman L (1985) Involvement of helicase II (uvrD gene product) and DNA polymerase I in excision mediated by the uvrABC protein complex. Proc Natl Acad Sci U S A 82: 4925-4929. doi:10.1073/pnas.82.15.4925. PubMed: 3161077. 3161077 PMC390470

[11] Maluf NK, Ali JA, Lohman TM (2003) Kinetic mechanism for formation of the active, dimeric UvrD helicase-DNA complex. J Biol Chem 278: 31930-31940. doi:10.1074/jbc.M304223200. PubMed: 12788954. 12788954

[12] Maluf NK, Fischer CJ, Lohman TM (2003) A Dimer of Escherichia coli UvrD is the active form of the helicase in vitro. J Mol Biol 325: 913-935. doi:10.1016/S0022-2836(02)01277-9. PubMed: 12527299. 12527299

[13] Niedziela-Majka A, Chesnik MA, Tomko EJ, Lohman TM (2007) Bacillus stearothermophilus PcrA monomer is a single-stranded DNA translocase but not a processive helicase in vitro. J Biol Chem 282: 27076-27085. doi:10.1074/jbc.M704399200. PubMed: 17631491. 17631491

[14] Sun B, Wei KJ, Zhang B, Zhang XH, Dou SX et al. (2008) Impediment of E. coli UvrD by DNA-destabilizing force reveals a strained-inchworm mechanism of DNA unwinding. EMBO J 27: 3279-3287. doi:10.1038/emboj.2008.240. PubMed: 19008855. 19008855 PMC2609735

[15] Yokota H, Chujo YA, Harada Y (2013) Single-molecule imaging of the oligomer formation of the nonhexameric Escherichia coli UvrD helicase. Biophys J 104: 924-933. doi:10.1016/j.bpj.2013.01.014. PubMed: 23442971. 23442971 PMC3576528

[16] Subramanya HS, Bird LE, Brannigan JA, Wigley DB (1996) Crystal structure of a DExx box DNA helicase. Nature 384: 379-383. doi:10.1038/384379a0. PubMed: 8934527. 8934527

[17] Korolev S, Hsieh J, Gauss GH, Lohman TM, Waksman G (1997) Major domain swiveling revealed by the crystal structures of complexes of E. coli Rep helicase bound to single-stranded DNA and ADP. Cell 90: 635-647. doi:10.1016/S0092-8674(00)80525-5. PubMed: 9288744. 9288744

[18] Velankar SS, Soultanas P, Dillingham MS, Subramanya HS, Wigley DB (1999) Crystal structures of complexes of PcrA DNA helicase with a DNA substrate indicate an inchworm mechanism. Cell 97: 75-84. doi:10.1016/S0092-8674(00)80716-3. PubMed: 10199404. 10199404

[19] Lee JY, Yang W (2006) UvrD helicase unwinds DNA one base pair at a time by a two-part power stroke. Cell 127: 1349-1360. doi:10.1016/j.cell.2006.10.049. PubMed: 17190599. 17190599 PMC1866287

[20] Yang W (2010) Lessons learned from UvrD helicase: mechanism for directional movement. Annu Rev Biophys 39: 367-385. doi:10.1146/annurev.biophys.093008.131415. PubMed: 20192763. 20192763 PMC3480338

[21] Kabsch W (2010) Xds. Acta Crystallogr D Biol Crystallogr 66: 125-132. doi:10.1107/S0907444909047337. PubMed: 20124692. 20124692 PMC2815665

[22] Leslie AGW (1992) Recent changes to the MOSFLM package for processing film and image plate data. Joint CCP4 and ESF-EACMB Newsletter on Protein Crystallography No26 Warrington, UK: Daresbury Laboratory.

[23] Keegan RM, Winn MD (2007) Automated search-model discovery and preparation for structure solution by molecular replacement. Acta Crystallogr D Biol Crystallogr 63: 447-457. doi:10.1107/S0907444907002661. PubMed: 17372348. 17372348

[24] Emsley P, Cowtan K (2004) Coot: model-building tools for molecular graphics. Acta Crystallogr D Biol Crystallogr 60: 2004-2132. Epub 10.1107/S0907444904019158. PubMed: 15572765. 15572765

[25] McCoy AJ, Grosse-Kunstleve RW, Adams PD, Winn MD, Storoni LC et al. (2007) Phaser crystallographic software. J Appl Crystallogr 40: 658-674. doi:10.1107/S0021889807021206. PubMed: 19461840. 19461840 PMC2483472

[26] Murshudov GN, Vagin AA, Lebedev A, Wilson KS, Dodson EJ (1999) Efficient anisotropic refinement of macromolecular structures using FFT. Acta Crystallogr D Biol Crystallogr 55: 247-255. doi:10.1107/S090744499801405X. PubMed: 10089417. 10089417

[27] Afonine PV, Grosse-Kunstleve RW, Adams PD (2005) The Phenix refinement framework. CCP4. Newsletter 42

[28] DeLano WL (2002) The PyMOL Molecular Graphics System. DeLano Scientific.

[29] Pettersen EF, Goddard TD, Huang CC, Couch GS, Greenblatt DM et al. (2004) UCSF Chimera--a visualization system for exploratory research and analysis. J Comput Chem 25: 1605-1612. doi:10.1002/jcc.20084. PubMed: 15264254. 15264254

[30] Panuska JR, Goldthwait DA (1980) A DNA-dependent ATPase from T4-infected Escherichia coli. Purif PropertieS A 63: 000-dalton enzyme and its conversion to a 22,000-dalton form. J Biol Chem 255: 5208-5214. 6154701

[31] Wu Y, Sommers JA, Suhasini AN, Aggarwal M, Brosh RM (2010) Molecular analyses of DNA helicases involved in the replicational stress response. Methods 51: 303-312. doi:10.1016/j.ymeth.2010.02.021. PubMed: 20188837. 20188837 PMC2900471

[32] Morris PD, Tackett AJ, Raney KD (2001) Biotin-streptavidin-labeled oligonucleotides as probes of helicase mechanisms. Methods 23: 149-159. doi:10.1006/meth.2000.1116. PubMed: 11181034. 11181034

[33] LiCata VJ, Wowor AJ (2008) Applications of fluorescence anisotropy to the study of protein-DNA interactions. Methods Cell Biol 84: 243-262. doi:10.1016/S0091-679X(07)84009-X. PubMed: 17964934. 17964934

[34] Manelyte L, Guy CP, Smith RM, Dillingham MS, McGlynn P et al. (2009) The unstructured C-terminal extension of UvrD interacts with UvrB, but is dispensable for nucleotide excision repair. DNA Repair (Amst) 8: 1300-1310. doi:10.1016/j.dnarep.2009.08.005. PubMed: 19762288. 19762288 PMC2997466

[35] Kumar S, Wolfson HJ, Nussinov R (2001) Protein flexibility and electrostatic interactions. IBM J Res Dev 45: 499-512. doi:10.1147/rd.453.0499.

[36] Pace CN, Fu H, Fryar KL, Landua J, Trevino SR et al. (2011) Contribution of hydrophobic interactions to protein stability. J Mol Biol 408: 514-528. doi:10.1016/j.jmb.2011.02.053. PubMed: 21377472. 21377472 PMC3086625

[37] Sabarinathan R, Aishwarya K, Sarani R, Vaishnavi MK, Sekar K (2011) Water-mediated ionic interactions in protein structures. J Biosci 36: 253-263. doi:10.1007/s12038-011-9067-4. PubMed: 21654080. 21654080

[38] Matson SW, George JW (1987) DNA helicase II of Escherichia coli. Characterization of the single-stranded DNA-dependent NTPase and helicase activities. J Biol Chem 262: 2066-2076. PubMed: 3029063. 3029063

[39] Zhang W, Dillingham MS, Thomas CD, Allen S, Roberts CJ et al. (2007) Directional loading and stimulation of PcrA helicase by the replication initiator protein RepD. J Mol Biol 371: 336-348. doi:10.1016/j.jmb.2007.05.050. PubMed: 17574572. 17574572

[40] Unciuleac MC, Shuman S (2010) Double strand break unwinding and resection by the mycobacterial helicase-nuclease AdnAB in the presence of single strand DNA-binding protein (SSB). J Biol Chem 285: 34319-34329. doi:10.1074/jbc.M110.162925. PubMed: 20736178. 20736178 PMC2966045

[41] Suhasini AN, Brosh RM (2010) Mechanistic and biological aspects of helicase action on damaged DNA. Cell Cycle 9: 2317-2329. doi:10.4161/cc.9.12.11902. PubMed: 20574162. 20574162 PMC3032018

[42] Suhasini AN, Sommers JA, Mason AC, Voloshin ON, Camerini-Otero RD et al. (2009) FANCJ helicase uniquely senses oxidative base damage in either strand of duplex DNA and is stimulated by replication protein A to unwind the damaged DNA substrate in a strand-specific manner. J Biol Chem 284: 18458-18470. doi:10.1074/jbc.M109.012229. PubMed: 19419957. 19419957 PMC2709400

[43] Amaratunga M, Lohman TM (1993) Escherichia coli rep helicase unwinds DNA by an active mechanism. Biochemistry 32: 6815-6820. doi:10.1021/bi00078a003. PubMed: 8392863. 8392863

[44] Fischer CJ, Maluf NK, Lohman TM (2004) Mechanism of ATP-dependent translocation of E.coli UvrD monomers along single-stranded DNA. J Mol Biol 344: 1287-1309. doi:10.1016/j.jmb.2004.10.005. PubMed: 15561144. 15561144

[45] Tomko EJ, Fischer CJ, Lohman TM (2012) Single-stranded DNA translocation of E. coli UvrD monomer is tightly coupled to ATP hydrolysis. J Mol Biol 418: 32-46. doi:10.1016/j.jmb.2012.02.013. PubMed: 22342931. 22342931 PMC3311787

[46] Tomko EJ, Jia H, Park J, Maluf NK, Ha T et al. (2010) 5'-Single-stranded/duplex DNA junctions are loading sites for E. coli UvrD translocase. EMBO J 29: 3826-3839. doi:10.1038/emboj.2010.242. PubMed: 20877334. 20877334 PMC2989100

[47] Collins R, McCarthy TV (2003) Purification and characterization of Thermus thermophilus UvrD. Extremophiles 7: 35-41. PubMed: 12579378. 12579378 10.1007/s00792-002-0293-4

[48] Lohman TM, Chao K, Green JM, Sage S, Runyon GT (1989) Large-scale purification and characterization of the Escherichia coli rep gene product. J Biol Chem 264: 10139-10147. PubMed: 2524489. 2524489

[49] An L, Tang W, Ranalli TA, Kim HJ, Wytiaz J et al. (2005) Characterization of a thermostable UvrD helicase and its participation in helicase-dependent amplification. J Biol Chem 280: 28952-28958. doi:10.1074/jbc.M503096200. PubMed: 15955821. 15955821 PMC1361353

[50] Anand SP, Khan SA (2004) Structure-specific DNA binding and bipolar helicase activities of PcrA. Nucleic Acids Res 32: 3190-3197. doi:10.1093/nar/gkh641. PubMed: 15199167. 15199167 PMC434446

[51] Chang TL, Naqvi A, Anand SP, Kramer MG, Munshi R et al. (2002) Biochemical characterization of the Staphylococcus aureus PcrA helicase and its role in plasmid rolling circle replication. J Biol Chem 277: 45880-45886. doi:10.1074/jbc.M207383200. PubMed: 12244110. 12244110

[52] Constantinesco F, Forterre P, Koonin EV, Aravind L, Elie C (2004) A bipolar DNA helicase gene, herA, clusters with rad50, mre11 and nurA genes in thermophilic archaea. Nucleic Acids Res 32: 1439-1447. doi:10.1093/nar/gkh283. PubMed: 14990749. 14990749 PMC390275

[53] Naqvi A, Tinsley E, Khan SA (2003) Purification and characterization of the PcrA helicase of Bacillus anthracis. J Bacteriol 185: 6633-6639. doi:10.1128/JB.185.22.6633-6639.2003. PubMed: 14594837. 14594837 PMC262108

[54] Soultanas P, Dillingham MS, Papadopoulos F, Phillips SE, Thomas CD et al. (1999) Plasmid replication initiator protein RepD increases the processivity of PcrA DNA helicase. Nucleic Acids Res 27: 1421-1428. doi:10.1093/nar/27.6.1421. PubMed: 10037801. 10037801 PMC148333

[55] Atkinson J, Guy CP, Cadman CJ, Moolenaar GF, Goosen N et al. (2009) Stimulation of UvrD helicase by UvrAB. J Biol Chem 284: 9612-9623. doi:10.1074/jbc.M808030200. PubMed: 19208629. 19208629 PMC2666613

[56] Zhang G, Deng E, Baugh L, Kushner SR (1998) Identification and characterization of Escherichia coli DNA helicase II mutants that exhibit increased unwinding efficiency. J Bacteriol 180: 377-387. PubMed: 9440527. 9440527 10.1128/jb.180.2.377-387.1998 PMC106893

[57] Cheng W, Brendza KM, Gauss GH, Korolev S, Waksman G et al. (2002) The 2B domain of the Escherichia coli Rep protein is not required for DNA helicase activity. Proc Natl Acad Sci U S A 99: 16006-16011. doi:10.1073/pnas.242479399. PubMed: 12441398. 12441398 PMC138555

[58] Jia HF, Korolev S, Niedziela-Majka A, Maluf NK, Gauss GH et al. (2011) Rotations of the 2B Sub-domain of E-coli UvrD Helicase/Translocase Coupled to Nucleotide and DNA Binding. J Mol Biol 411: 633-648. doi:10.1016/j.jmb.2011.06.019. PubMed: 21704638. 21704638 PMC3146578

[59] Cao Z, Julin DA (2009) Characterization in vitro and in vivo of the DNA helicase encoded by Deinococcus radiodurans locus DR1572. DNA Repair (Amst) 8: 612-619. doi:10.1016/j.dnarep.2008.12.011. PubMed: 19179120. 19179120

[60] Dillingham MS (2011) Superfamily I helicases as modular components of DNA-processing machines. Biochem Soc Trans 39: 413-423. doi:10.1042/BST0390413. PubMed: 21428912. 21428912

