# Supplementary material for: Structural and Mechanistic Insight into DNA Unwinding by Deinococcus radiodurans UvrD
Source: PLoS One. 2013 Oct 15;8(10):e77364. doi: 10.1371/journal.pone.0077364 (PMC3797037; doi:10.1371/journal.pone.0077364)
Supplement: Table S1 — Sequences of DNA oligonucleotides used in this study. (DOCX) [file pone.0077364.s001.docx]

**Table S1**

| **Name** | **Sequence** |
| --- | --- |
| For25 | 5′-GCAGTGCTCGCAGGTCGTTTTTTTT-3′ |
| Rev25 | 5′-ACGACCTGCGAGCACTGCTTTTTTT-3′ |
| For28 | 5′-GCAGTGCTCGCAGGTCGTACCTTTTTTT-3′ |
| Rev28 | 5′-GGTACGACCTGCGAGCACTGCTTTTTTT-3′ |
| For25-21F | 5′-GCAGTGCTCGCAGGTCGTTT**F**TTTT-3′ |
| Rev25-21F | 5′-ACGACCTGCGAGCACTGCTT**F**TTTT-3′ |
| H1 | 5′-TCGGTACGACCTGCGAGCACTGCTT-3′ |
| H1T12 | 5'-TCGGTACGACC**F**GCGAGCACTGCTT-3' |
| H1-3F | 5′-TCGGTACGACCTGCGAGCACTGCTT-**Fluo**-3′ |
| H1-5FAM | 5′-**FAM**-TCGGTACGACCTGCGAGCACTGCTT-3′ |
| H3-15 | 5′-AAGCAGTGCTCGCAGGTCGTACCGATTTTTTTTTTTTTTT-3′ |
| H3-7 | 5'-AAGCAGTGCTCGCAGGTCGTACCGATTTTTTT-3' |
| H4 | 5′-AAGCAGTGCTCGCAGGTCGTACCGA-3′ |
| H5-15 | 5′-TTTTTTTTTTTTTTTAAGCAGTGCTCGCAGGTCGTACCGA-3′ |
| H5-7 | 5'-TTTTTTTAAGCAGTGCTCGCAGGTCGTACCGA-3' |
| H3-25-B49 | 5'-AAGCAGTGCTCGCAGGTCGTACCGATTTTTTTTTTTTTTTTTTTTTTT**B**T-3' |
| H5-25-B2 | 5'-T**B**TTTTTTTTTTTTTTTTTTTTTTTAAGCAGTGCTCGCAGGTCGTACCGA-3' |

**F** indicates the position of the fluorescein-conjugated thymine

**Fluo** stands for fluorescein

**FAM** stands for fluorescein amidite

**B** indicates the position of biotin-conjugated thymine
