# Supplementary material for: Structural and Mechanistic Insight into DNA Unwinding by Deinococcus radiodurans UvrD
Source: PLoS One. 2013 Oct 15;8(10):e77364. doi: 10.1371/journal.pone.0077364 (PMC3797037; doi:10.1371/journal.pone.0077364)
Supplement: Table S2 — Nature of contacts between the various domains of nucleotide-bound ec- and drUvrD. (DOCX) [file pone.0077364.s002.docx]

**Table S2**

| **Domains** | **Interface Area (Å^2^)** | | **Hydrogen bonds** | | **Salt bridges** | |
| --- | --- | --- | --- | --- | --- | --- |
|  | *ec*UvrD | *dr*UvrD | *ec*UvrD | *dr*UvrD | *ec*UvrD | *dr*UvrD |
| 1A-1B | 1221 | 1141 | 12 | **18** | 0 | **4** |
| 1A-2A | 1114 | 1034 | 19 | 20 | 10 | 8 |
| 1B-2B | 1036 | **713** | 6 | **12** | 9 | **22** |
