# Supplementary material for: Structural and Mechanistic Insight into DNA Unwinding by Deinococcus radiodurans UvrD
Source: PLoS One. 2013 Oct 15;8(10):e77364. doi: 10.1371/journal.pone.0077364 (PMC3797037; doi:10.1371/journal.pone.0077364)
Supplement: Table S3 — Summary of the helical parameters of the DNA duplexes bound to drUvrD compared to ideal B-form DNA. (DOCX) [file pone.0077364.s003.docx]

**Table S3**

|  |  | Helical Twist (°) | Nucleotide/pitch | Axis-bend (°) |
| --- | --- | --- | --- | --- |
| 1 | *dr*UvrD^FL^ | 34.8 | 10.3 | 25.3 |
| 2 | *dr*UvrD^∆C^ form I | 35.6 | 10.1 | 16.5 |
| 3 | *dr*UvrD^∆C^ form II | 33.4 | 10.8 | 11.6 |
| 4 | Ideal B DNA | 36.0 | 10.0 | 0.1 |
