# Supplementary figures and images for: Structural and Mechanistic Insight into DNA Unwinding by Deinococcus radiodurans UvrD
Source: PLoS One. 2013 Oct 15;8(10):e77364. doi: 10.1371/journal.pone.0077364 (PMC3797037; doi:10.1371/journal.pone.0077364)

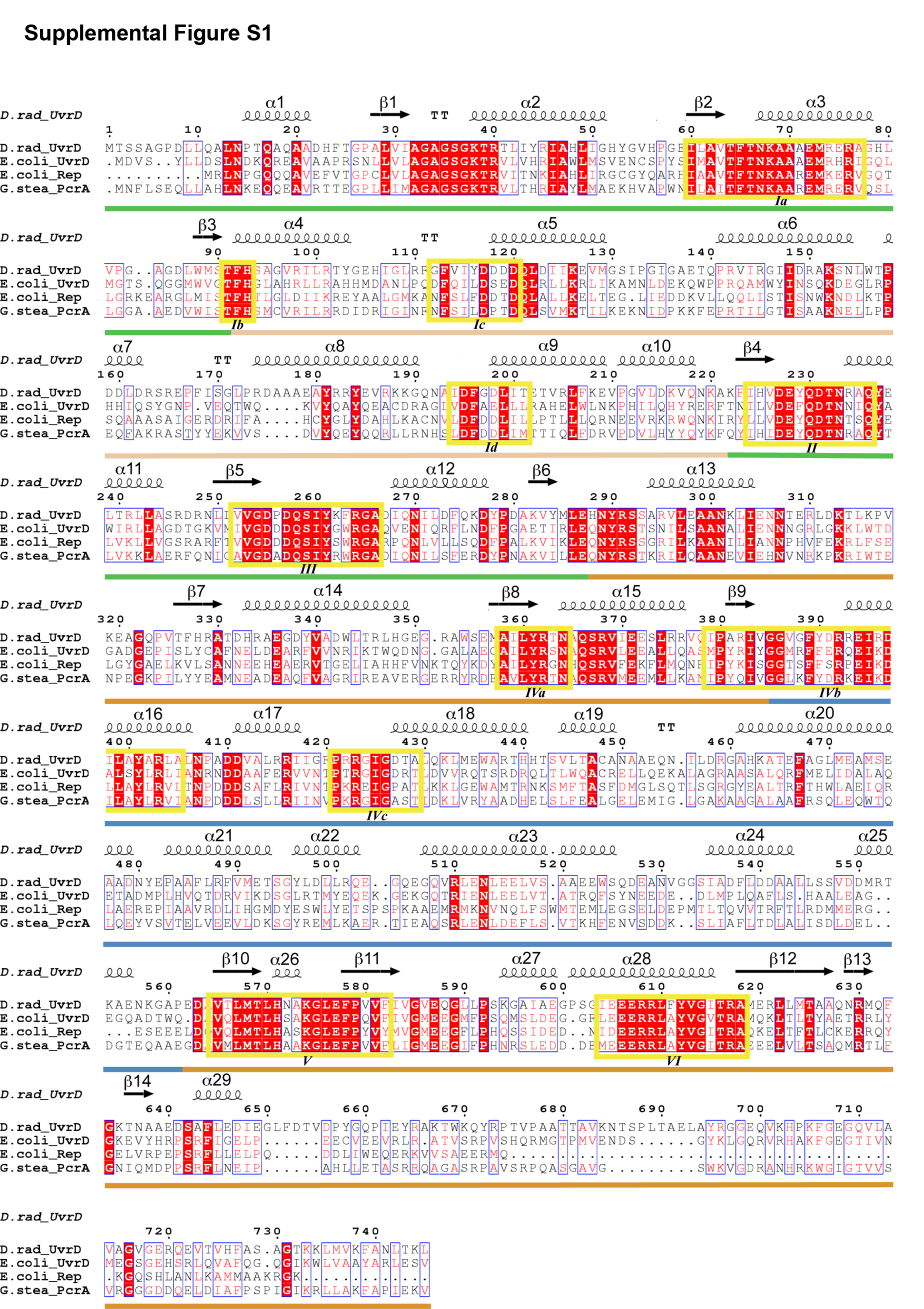

Supplement: Figure S1 — Sequence alignment of D. radiodurans UvrD, E. coli UvrD, E. coli Rep and G. stearothermophilus PcrA helicases. The secondary structure of drUvrD is shown above the alignment and the domains are illustrated as colored lines below the alignment. The domains are colored as in Figure 1. The conserved helicase motifs are numbered and marked with yellow boxes. (TIF) [file pone.0077364.s005.tif]

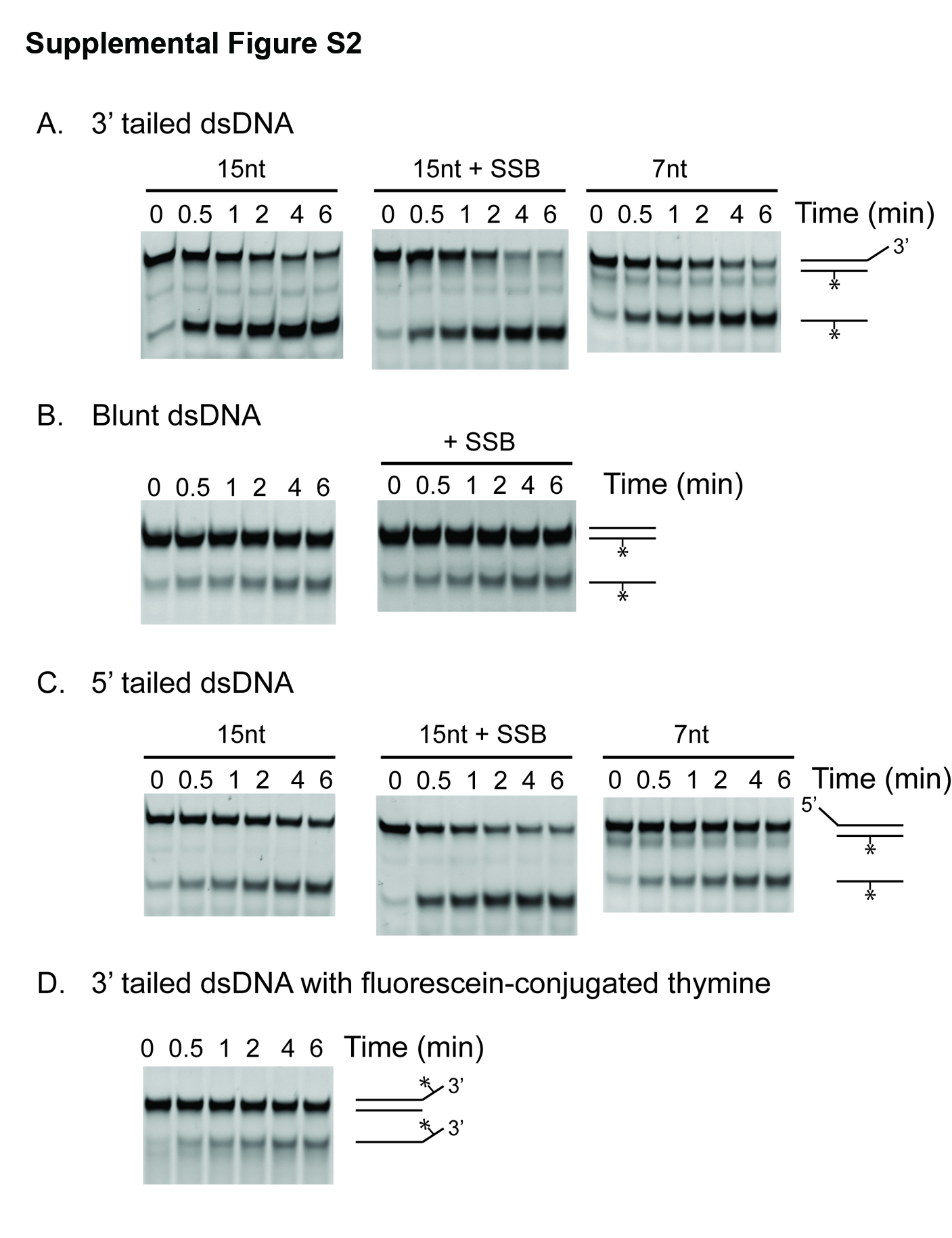

Supplement: Figure S2 — Helicase activity on 3′-, 5′-tailed and blunt dsDNA. A. drUvrD (250nM) unwinding of 3′-tailed 25 base-pair dsDNA (20nM) with either 15nt- or 7nt ssDNA extensions in the absence and presence of SSB (250nM). B. drUvrD (250nM) unwinding of blunt 25 base-pair dsDNA (20nM) in the absence and presence of SSB (250nM). C. drUvrD (250nM) unwinding of 5′-tailed 25 base-pair dsDNA (20nM) with either 15nt- or 7nt ssDNA extensions in the absence and presence of SSB (250nM). A-C. Reactions were stopped at the following time points: 0, 30sec, 1min, 2min, 4min and 6min, prior to separation on 20% TBE gels. The fluorescein label is illustrated as a star in the schematic representation of the DNA. (TIF) [file pone.0077364.s006.tif]

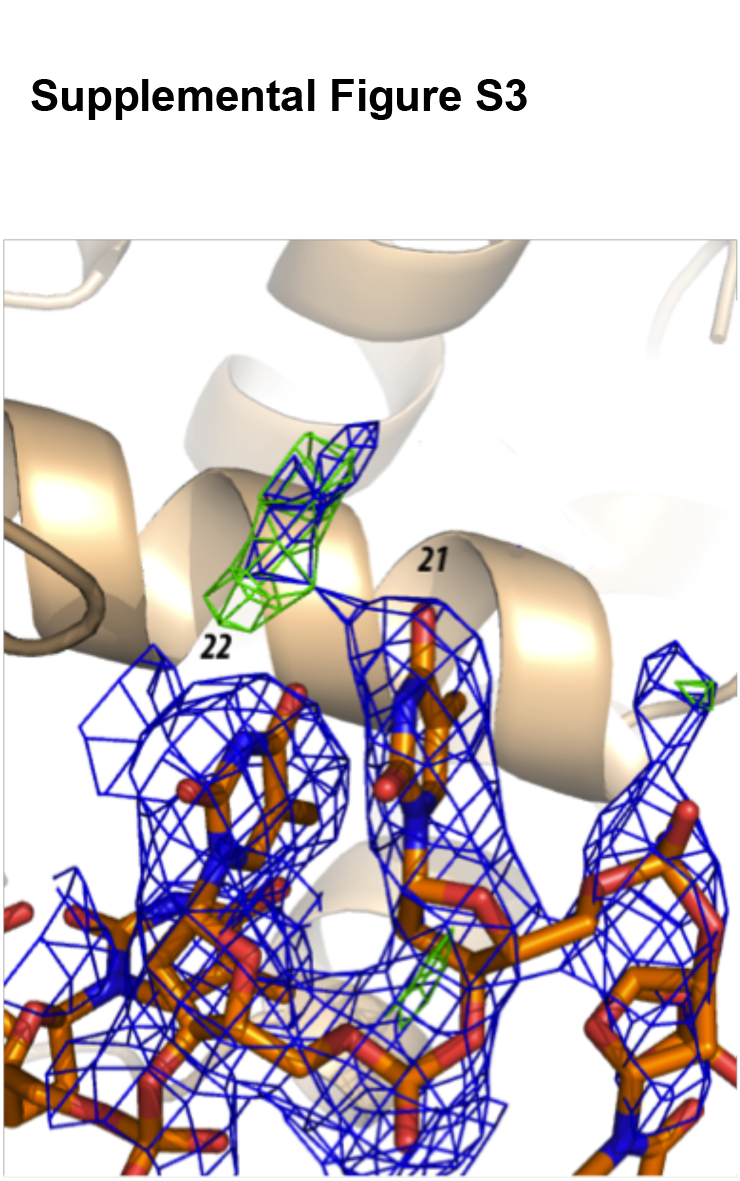

Supplement: Figure S3 — Binding of fluorescein-labeled DNA to drUvrD∆C. The DNA oligonucleotides contain a fluorescein-conjugated thymine at position 21 within the ssDNA extension. The 2Fo-Fc electron density map (blue) is contoured at 1σ, while the Fo-Fc difference density map (green) is contoured at 2.5σ. The ssDNA is illustrated in sticks. (TIF) [file pone.0077364.s007.tif]

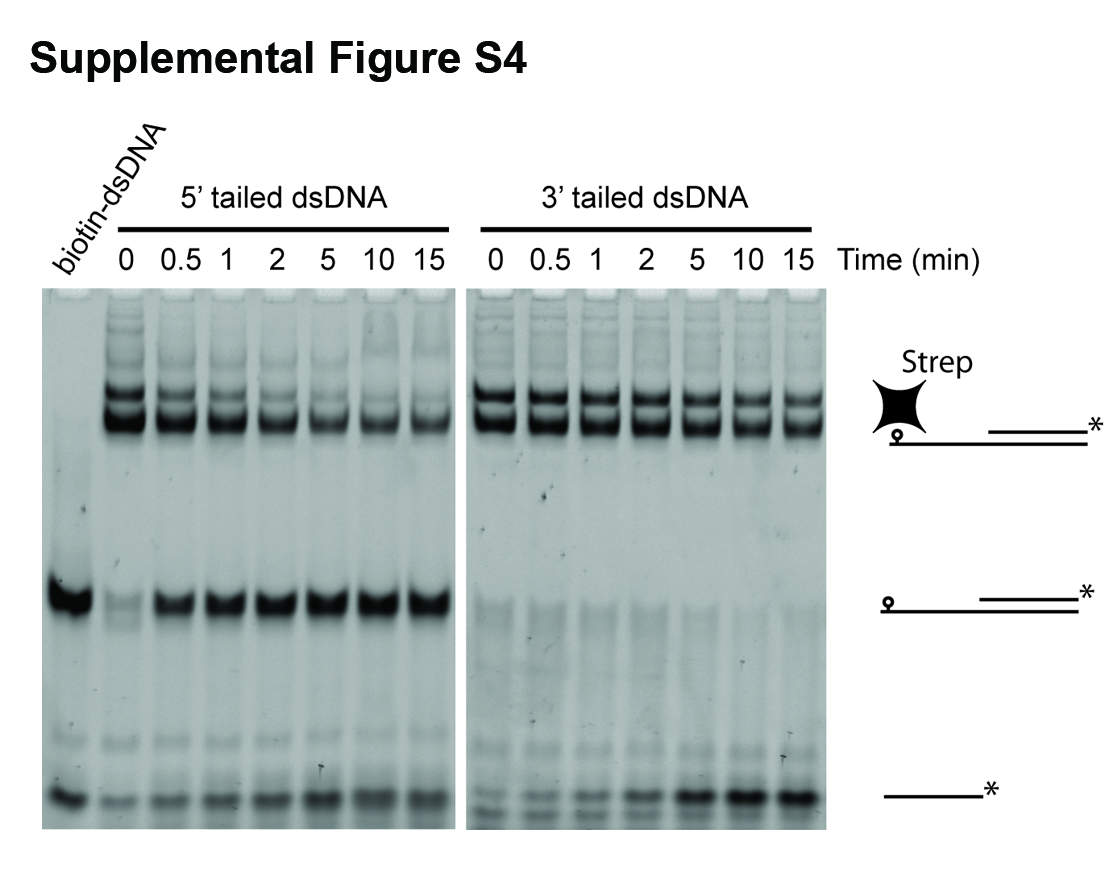

Supplement: Figure S4 — Translocase activity on 3′- and 5′-tailed dsDNA. drUvrD (250nM) translocation activity on streptavidin bound 5′- and 3′-tailed 25 base-pair dsDNA (20nM) with 25nt ssDNA extensions. Reactions were stopped at the following time points: 0, 30sec, 1min, 2min, 5min, 10 min and 15min, prior to separation on 10% TBE gels. The fluorescein and the biotin labels are illustrated respectively as a star and an open circle in the schematic representation of the DNA. The upper bands correspond to streptavidin-bound dsDNA substrate, the middle-band to the released dsDNA (translocase product) and the lower band corresponds to the product of the helicase activity of UvrD, i.e. ssDNA. Biotinylated dsDNA without streptavidin was loaded in the first well. (TIF) [file pone.0077364.s008.tif]

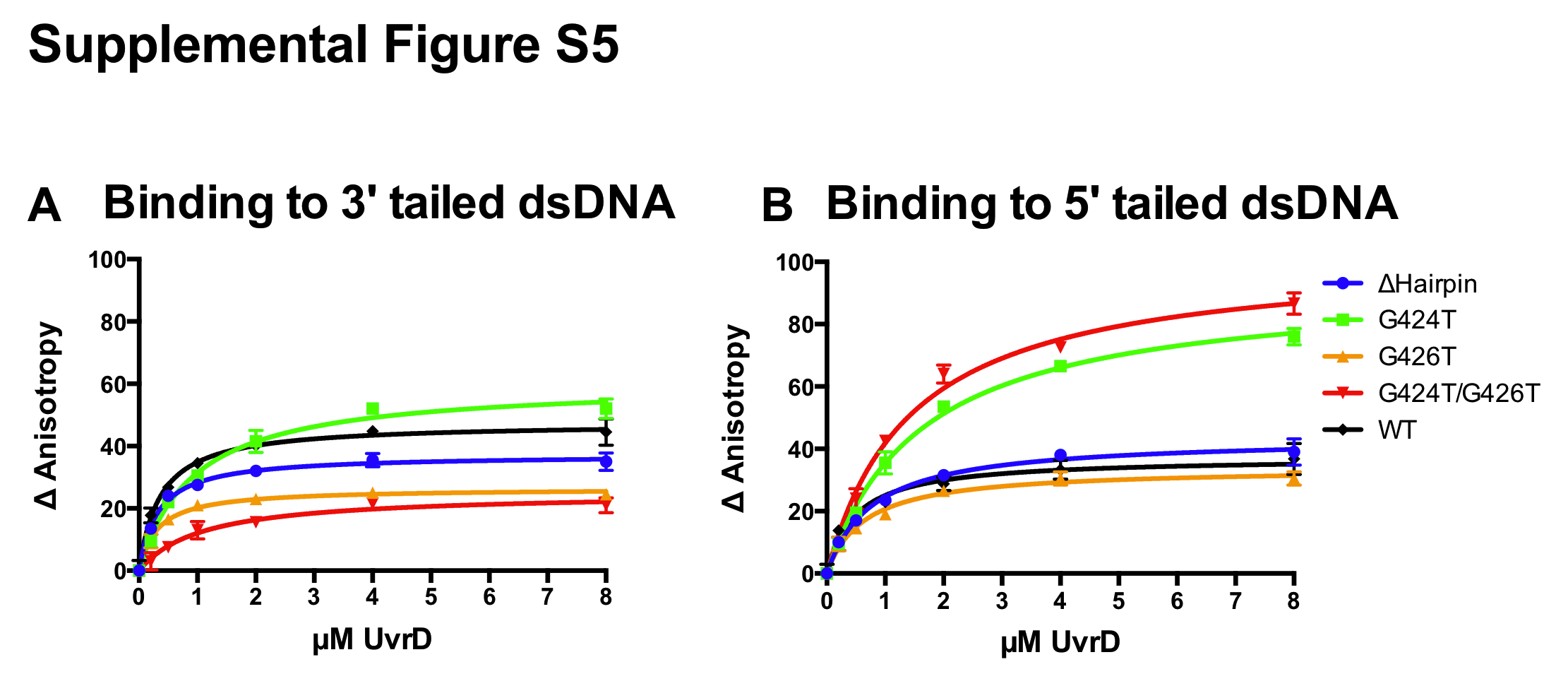

Supplement: Figure S5 — DNA binding to 3′- and 5′-tailed dsDNA. Binding of wild-type (WT) and mutant drUvrD to 3′- (A) and 5′-tailed dsDNA (B) was measured by fluorescence anisotropy. The anisotropy measured for the DNA alone was subtracted from all other values and the change in anisotropy (ΔA) is plotted as a function of UvrD concentration (µM). The averaged data points were fitted to a standard binding equation assuming a single binding site using GraphPad Prism6. Standard deviations are shown as vertical bars. (TIFF) [file pone.0077364.s009.tiff]
